# Epidemiological Determinants of Patient Non-Conveyance to the Hospital in an Emergency Medical Service Environment

**DOI:** 10.3390/ijerph20146404

**Published:** 2023-07-20

**Authors:** Hassan Farhat, Cyrine Abid, Kawther El Aifa, Padarath Gangaram, Andre Jones, Mohamed Chaker Khenissi, Moncef Khadhraoui, Imed Gargouri, Loua Al-Shaikh, James Laughton, Guillaume Alinier

**Affiliations:** 1 Ambulance Service, Hamad Medical Corporation, Doha P.O. Box 3050, Qatar; 2 Faculty of Sciences, University of Sfax, Sfax P.O. Box 3000, Tunisia; 3 Faculty of Medicine “Ibn El Jazzar”, University of Sousse, Sousse P.O. Box 4000, Tunisia; 4 Faculty of Medicine, University of Sfax, Sfax P.O. Box 3000, Tunisia; 5 Faculty of Health Sciences, Durban University of Technology, P.O. Box 1334, Durban 4000, South Africa; 6 Higher Institute of Biotechnology, University of Sfax, Sfax P.O. Box 3038, Tunisia; 7 School of Health and Social Work, University of Hertfordshire, Hatfield AL10 9AB, UK; 8 Weill Cornell Medicine-Qatar, Doha P.O. Box 24144, Qatar; 9 Faculty of Health and Life Sciences, Northumbria University, Newcastle upon Tyne NE1 8ST, UK

**Keywords:** non-conveyance, emergency medical services, paramedics, Middle East, pre-hospital environment

## Abstract

Background: The increasing prevalence of comorbidities worldwide has spurred the need for time-effective pre-hospital emergency medical services (EMS). Some pre-hospital emergency calls requesting EMS result in patient non-conveyance. Decisions for non-conveyance are sometimes driven by the patient or the clinician, which may jeopardize the patients’ healthcare outcomes. This study aimed to explore the distribution and determinants of patient non-conveyance to hospitals in a Middle Eastern national Ambulance Service that promotes the transportation of all emergency call patients and does not adopt clinician-based non-conveyance decision. Methods: Using R Language, descriptive, bivariate, and binary logistic regression analyses were conducted for 334,392 multi-national patient non-conveyance emergency calls from June 2018 to July 2022, from a total of 1,030,228 calls to which a response unit was dispatched. Results: After data pre-processing, 237,862 cases of patient non-conveyance to hospital were retained, with a monthly average of 41.96% (*n* = 8799) of the emergency service demands and a standard deviation of 5.49% (*n* = 2040.63). They predominantly involved South Asians (29.36%, *n* = 69,849); 64.50% (*n* = 153,427) were of the age category from 14 to 44 years; 61.22% (*n* = 145,610) were male; 74.59% (*n* = 177,424) from the urban setting; and 71.28% (*n* = 169,552) had received on-scene treatment. Binary logistic regression with full variables and backward methods identified the final models of the determinants of patient non-conveyance decisions with an Akaike information criterion prediction estimator, respectively, of (250,200) and (250,169), indicating no significant difference between both models (Chi-square test; *p*-value = 0.63). Conclusions: Despite exercising a cautious protocol by encouraging patient transportation to hospital, patient non-conveyance seems to be a problem in the healthcare system that strains the pre-hospital medical response teams’ resources. Policies and regulations should be adopted to encourage individuals to access other primary care centers when required rather than draining emergency services for non-emergency situations.

## 1. Introduction

Pre-hospital emergency medical services (EMS) provide medical assistance to the sick and injured when an emergency call is initiated. Once a patient, bystander, or next-of-kin makes an emergency call, an EMS team will immediately be dispatched to rapidly reach the patient, provide emergency treatment, and transport them to hospital [1]. Research has shown that efficient pre-hospital medical assistance increases the chance of pre-hospital survival [2]. However, not all emergency responses result in a patient being conveyed to hospital. Research defined the non-conveyance to the hospital by discharging a patient after a successful evaluation on the scene [3,4]. Other research defined patient non-conveyance to hospital as EMS missions resulting in non-transport based on the patient’s decision [5]. Hence, for various reasons, the percentage of patient non-conveyance varies between countries (3.7–93.7%) [6]. The non-conveyance decision by the patient or their next of kin may result in subsequent adverse health outcomes, jeopardize the patient’s condition, and delay health recovery [7]. In addition, non-conveyance decisions burden the EMS resource allocation and availability. However, it may alleviate the demands on healthcare facilities. EMS assistance is sometimes requested for minor medical or trauma complaints that do not necessarily require further care and transport to hospital. Subsequently, this might compromise EMS availability to respond to other time-critical life-threatening emergencies. To our knowledge, very few studies have been conducted which explore patient non-conveyance by EMS in the Middle East. A recent study reported that patient non-conveyance represents 34.4% of the emergency demand in Saudi Arabia [8].

Qatar is a Middle Eastern country with a multi-national population of different ethnic groups, with a dominance of South Asians and Arabs, including Qataris [9,10]. The population is predominantly constituted of males [9], in relation to the ongoing development of the country’s infrastructures. Further, Hamad Medical Corporation Ambulance Service (HMCAS) is Qatar’s only governmental pre-hospital EMS [11]. HMCAS is a modern ambulance service that provides free pre-hospital emergency care to all Qatar residents, citizens, and visitors [12,13]. HMCAS also has a non-emergency service which provides inter-facility transfers nationally and internationally. Emergency services consist of paramedics and critical care paramedics responding to 999 emergency calls within the community in Qatar or emergency departments of governmental and private healthcare facilities. HMCAS promotes patient transport to hospital for all patients they see unless the patient or next-to-kin (in case of pediatric or mental disability) refuses and signs the electronic refusal form. HMCAS does not adopt the practice of non-conveyance by clinician decisions and mandates to avoid suggesting or implying that transport to a healthcare facility might not be necessary for a patient. 

Once a call for service (CFS) is received through 999, and the location is identified, the Emergency Medical Dispatcher (EMD), who operates from the National Command Center (NCC), ensures the dispatch of the closest ambulance to the patient so they can be reached without delay [1]. Using computer-aided dispatch software, EMDs navigate various steps in a pre-determined international program called “ProQA” to determine the dispatch code according to the chief complaint and provide adequate pre-arrival instructions to the caller [14,15]. Then, using the Medical Priority Dispatch System (MPDS), the EMD determines the most appropriate resources to be dispatched. Once the ambulance arrives at the emergency scene, and the patient is clinically stabilized, paramedics are required to transport them to the most appropriate healthcare facility. However, upon receiving emergency treatment at the scene, some patients refuse to be transported to hospital, which can cause a delay in them receiving definitive therapeutic care and potentially jeopardize their prognosis in some cases [16]. The non-conveyance decision made by the patient or the next of kin can prevent patients with pre-existing comorbidities from receiving proper assessment and treatment from a specialist in a healthcare facility and increase the risk of severe harmful outcomes [17].

Additionally, the patient non-conveyance decision is challenging for HMCAS personnel, although their opinion has not been fully studied before, other than concerning a specific patient group who were identified as generating ambulance callbacks within a relatively short period of time for the same complaint (i.e., diabetes) [7]. Convincing a patient with a non-urgent minor medical problem to be conveyed to hospital may pose certain challenges. In a multi-national and linguistic population, language barriers may challenge conveyance decisions. Cultural and religious beliefs may also hamper conveyance to hospital [18]. The lack of access to healthcare resources or misunderstanding of freely available or chargeable healthcare services may also influence non-conveyance decisions. For example, patients who refuse transportation should be encouraged to visit primary healthcare centers, which are functional during the day and until late night hours, rather than calling 999 [11]. Presenting to a crowded emergency department (ED) with a patient who has a minor chief complaint exposes the EMS personnel to long “unnecessary” waits for the handover until further admissions to the ED are permitted [19,20,21]. Ethical issues further challenge the non-conveyance decision by EMS personnel due to potential associated risks [22]. Hence, HMCAS does not practice clinician-based patient non-conveyance. 

Consequently, the non-conveyance of patients from an emergency scene to a definitive care facility may impact the patient and the EMS system. The patient non-conveyance decisions might affect the overall healthcare system quality, as serious medical outcomes may result from a transport refusal decision by the patient [23]. Conversely, considerable resources might be wasted on dispatching EMS to respond to minor medical or trauma cases that do not require emergency or specialized pre-hospital medical care or transport to hospital. Research in England has suggested that profound and focused investigation strategies are needed to identify the dimensions of such decisions to formulate appropriate corrective strategies [24,25].

The descriptive and analytical epidemiology of patient non-transport decisions at HMCAS has not been studied previously and is thus poorly understood. Understanding the determinants and root causes will help build robust strategies to manage this issue effectively.

This retrospective quantitative analysis study investigated 999 emergency calls that resulted in patient non-conveyance to hospital decisions in a leading Middle Eastern pre-hospital EMS system.

## 2. Methods

### 2.1. Study Design and Setting

In this study, we conducted a retrospective quantitative analysis examining 999 emergency calls between 1 June 2018 and 31 July 2022 that ended with non-conveyance to hospital. The data were extracted from the “NJM” system in the National Command Centre (NCC) and the electronic Patient Care Record (ePCR) system managed by the HCMAS Business Intelligence (BI) department. NJM is the name derived from the Arabic name (النظام الجغرافي الموحد: نجم) of the computer-aided dispatch system that creates CFSs, controlled by the Minister of Interior and developed in Qatar [1].

### 2.2. Participants and Sampling

The inclusion criteria were defined as emergency calls received on 999 resulting in patient non-conveyance to hospital, as well as the following:

- Emergency calls cancelled by the caller before the paramedics’ arrival, or the caller was not found or did not answer the callback, as they result in the EMD engaging with a 999-emergency call processing and providing the ambulance pre-arrival instructions until the ambulance arrived on the scene and was later assigned as available;
- Paramedics arrived at the patient’s side and assessed them, but they refused transport;
- A call was received from an emergency department from another healthcare facility, but the patient refused to be transported to hospital and was released to the community.

The patient non-conveyance to hospital decisions was divided in the e-PCR system into three groups. These categories were pre-defined in the non-conveyance decision section of the ePCR system as approved by the HMCAS BI executive team. They consist of: (1) “Refused transport and not treated at the scene”, (2) “Refused transport but was treated at the scene”, and (3) “Death on arrival (DOA)”.

**Figure 1:**
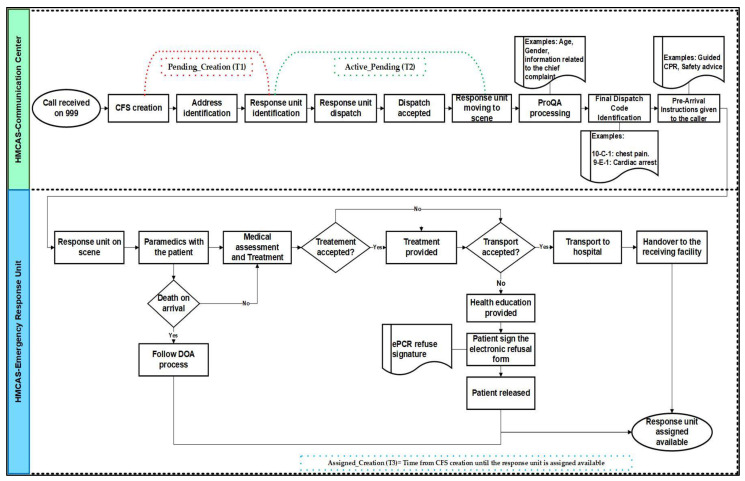
999 Emergency Call Management Process in the HMCAS.

Additionally, in Qatar, HMCAS response units do not usually transport a patient determined to be deceased upon their arrival (undeniable death as per HMCAS Clinical Practice Guidelines (CPG)). Ambulances were dispatched to cases later determined DOA, released when the police if needed, have completed their investigations, and then they have to follow the DOA case process. In this study, DOA was excluded in the bivariate and multivariate analyses and only included as a separate category in the descriptive analysis since they were processed through ProQA to generate the dispatch code. EMDs and paramedics were engaged with these calls from the emergency call time until the dispatched unit was assigned available (Figure 1). Figure 1 explains the process of 999 emergency call management in HMCAS. The patients’ identifiers were concealed by the HMCAS BI team using special codes.

### 2.3. Quantitative Variables Handling and Measurements

Fourteen quantitative and qualitative variables were studied. They were gender, age, zones (urban and rural), response call priorities (as determined by the MPDS: P1; with lights and sirens: P2: no lights, no siren, and the other priorities included the calls when a patient walked to the ambulance’s stand-by point), nationalities, chief complaints (during the call-taking), response unit, provisional diagnoses, response timings duration, the year and the month of the case.

Initial data pre-processing was conducted using R-programming Language accessed through the R-Studio environment to ensure the integrity and accuracy of the analysis (Supplementary files). Pre-processing consisted of improving big data and rendering these data more suitable for analysis. First, data transformation was carried out by converting the pre-existing data into a more understandable format for analysis [26]. For example, the response time durations were determined from the initial times provided in the dataset:

- T1: “Pending_Creation”: From when the EMD receives the emergency call until the closest unit to the emergency is found.
- T2: “Active_Pending”: From when the EMD finds the closest unit to dispatch until that unit accepts the allocation and starts moving to the emergency call location.
- T3: “Assigned_Creation”: From the time the EMD receives the emergency call until the unit dispatched treats the patient and is assigned as being available and ready for the next call.

Second, the variables were recoded as follows (Supplementary Materials):

- Age: Patients were divided into groups according to World Health Organization age categories [16] (≤14 years; (15–29); (30–44); (45–59); (60–74); (75–89) and ≥90).
- Nationalities: 273 nationalities, as recorded in the ePCR system. As the ePCR system has been evolving since it was implemented in the HMCAS in 2016 [27], each nationality was recorded in different ways (e.g.,: US, USA, and American). All recorded nationalities were grouped according to their geographical distribution [28]: Qatar, Gulf Cooperation Council (GCC), Middle East and North Africa (MENA), South Asia, East Asia and Pacific, Europe and Central Asia, Sub-Saharan Africa, North America, Latin America and the Caribbean, others, and unknown.
- Zones: The Ministry of the Municipality in Qatar divides the location into 98 pre-determined zones. They were grouped into urban and rural areas.
- Chief complaints: 1176 ProQA codes determined by the EMDs were grouped into 37 groups according to their chief complaints protocol defined by the International Academy of Emergency Medical Dispatchers [29].
- Response unit: 537 units were dispatched as determined by the MDPS:
  ▪ Advanced critical care response vehicle staffed with a critical care-credentialed paramedic and a critical care assistant (Charlie) [30,31].
  ▪ Ambulances and rapid response cars with ambulance paramedic-credentialed staff from the emergency section (Alpha, Bravo, Delta, Specialized Emergency Management (SEM) and Events units).
  ▪ Non-emergency section units with ambulance paramedic-credentialled staff (Foxtrot, Tango, COVID and Green bus). These can be dispatched if there is a lack of response units available in the emergency section at peak times, according to HMCAS Standard Operating Procedures.
- Provisional Diagnosis: 158 provisional diagnoses determined by paramedics were grouped into 33 groups as follows: allergic reaction, anaphylaxis, animal bite, burns, cardiac arrest, cardiovascular, respiratory, shock, chronic medical condition (CMC), neurological, gastro-intestinal-gastro-urinary (GIGU), endocrinology, obstetrics and gynecology (OBS GYN), combative patient, diabetic problem, COVID-19 related, febrile illness, heat-related, hazardous material (HazMat), toxicology (other than HazMat exposure), interfacility transport (IFT), minor illness, electrocution, epistaxis, trauma, minor trauma, near-drowning, non-specific-problems (NSP), pain, non-traumatic back-pain, parental concern, sick person, undeniable death (as defined by the HMCAS Clinical Practical Guidelines), and not recorded.
- Response timing duration (T1, T2, and T3).

14.01% (*n* = 46,848) of observations with missing values in the dataset were identified (One observation can have more than one missing value) and deleted, minimizing their already low weight in the dataset and reducing their negative effect on the analysis [23]. The unnecessary variables in the analysis were also removed from the dataset [24].

During the analysis, a code in R was determined to combine and read the different files received from the HMCAS BI team as a single unified file. Initially, the data were retrieved in separate files from different sources (NJM and ePCR) with large sizes for this study.

### 2.4. Statistical Analysis

First, descriptive statistics were determined to provide an overview of the general population studied. These included demographic information, response timing distribution, chief complaints, and provisional diagnosis distributions according to the determined age groups.

Second, mosaic plots were designed to help analyze the association level between two or more categorical variables through the residual value and provide a way to visualize the relationships between the categorical variables [25]. The higher the standard residuals’ value, the stronger the association between the categorical variables.

Third, a bivariate analysis was conducted. The Chi-square test was used to assess the relationship between the categorical variables [32]. Consequently, the following hypotheses were tested:

**Hypothesis 0 (H0).**  *There is no significant association between the categorical variables and the non-conveyance groups*.

**Hypothesis 1 (H1).**  *There is a significant association between the categorical variables and the non-conveyance groups*.

Additionally, the Mann-Whitney U test (also known as Wilcoxon rank sum) was conducted to assess if there is a difference between the independent groups by assessing the sum of ranks [32].

Therefore, the following hypotheses were tested:

**Hypothesis 2 (H2).**  *There is no difference between the groups*.

**Hypothesis 3 (H3).**  *There is a significant difference between the groups*.

Statistical multivariate modelling using binary logistic regression was also conducted to predict non-conveyance decisions based on the predictors mentioned in this study [33]. The full variables and backward methods were assessed [34]. The categorical outcomes were refused transport and not treated and treated and refused transport.

DOA and 999 emergency calls cancelled by the caller before the paramedics arrived, or the caller was not found or did not answer the callback, were not included in the bivariate and regression analyses.

The ethical review board of the Hamad Medical Corporation Medical Research Committee approved this study under reference number MRC-01-22-264.

## 3. Results

From January 2018 to July 2022, 334,392 patient non-conveyance cases were recorded and met the inclusion criteria out of 1,030,228 calls to which a response unit was dispatched. After removing the monthly data with significantly reduced non-conveyance numbers (outliers due to a technical problem with the archiving system) from both non-conveyance and total emergency demand call numbers, the monthly average percentage of non-conveyance was determined. It was equal to 41.95% (*n* = 8799) of the emergency service demands, with a monthly standard deviation equal to 5.49% (*n* = 2040.63) of the calls received through 999. After data pre-processing and removal of 14.01% (*n* = 46,848) of patients’ non-conveyance cases due to missing values, a total of 237,862 cases of non-conveyance to the hospital were retained. Furthermore, 1.03% (*n* = 3452) of the total patient non-conveyance calls were either cancelled by the EMD or because the caller was unavailable upon the ambulance’s arrival and had to be assigned as available for the next call. Furthermore, of the overall patient non-conveyance numbers, 71.28% (*n* = 169,552) did not require treatment on scene, 28.19% (*n* = 67,062) received treatment on scene, and 0.52% (*n* = 1248) were cases of DOA.

For the demographic information, 61.22% (*n* = 145,610) were male, whereas 38.78% (*n* = 92,252) were female. The 15–44-years age group was the prominent category, with 64.50% (*n* = 153,427) of the non-conveyance. A total of 74.59% (*n* = 177,424) of non-conveyance occurred in the urban zones, and 25.409% (*n* = 60,438) in the rural zones. For the age group ≤ 14 years, 10.41% (*n* = 24,769) of non-conveyance was recorded. Only 4.11% (*n* = 9779) of the patients were older than 74 years of age. Nationals of South Asian countries were the most represented, contributing to 29.37% (*n* = 69,849) of the total non-conveyance cases (Table 1).

**Figure 2:**
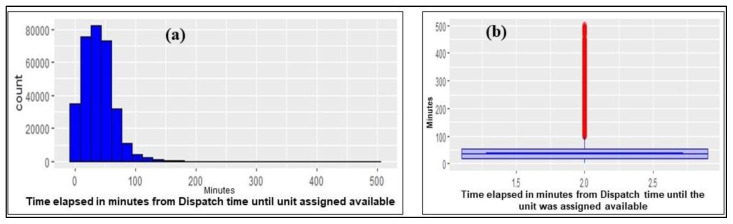
Distribution of non-conveyance times elapsed.

During the call-taking, the time that elapsed from when the EMD received the call until the emergency address and the closest unit to be dispatched were identified was less than one minute which is on par with international benchmarks [35]. The time elapsed from when the emergency response unit was dispatched until it was assigned available was around 60 min (Figure 2a,b). Rarely, such as in the case of a warehouse fire incident in an agricultural area where controlling the blaze took hours, an ambulance had to remain in position until Civil Defense controlled the scene, resulting in a prolonged response time. 

For the EMD call-taking protocols (P), Protocol 36 for the “Pandemic” and 26 for “sick person” were the major chief complaints, i.e., 16.58% (*n* = 39,447) and 11.39% (*n* = 27,100), respectively, of the whole non-conveyance rate (Table 2). Moreover, “Non-Specific-Problems” (NSP) 21.93% (*n* = 52,151) and “Pain” 15.12% (*n* = 35,975) were the most common provisional diagnoses after paramedic assessment (Table 3).

Likewise, the *p*- and X^2^ (a criterion used to assess how likely an observed difference between the actual frequencies in the data and the theoretical expectations if it is due to chance) values in Table 4 Part I indicated a strong association between most of the categorical variables and that the results were unlikely due to chance. Thus, the null hypothesis was rejected, and H_1_ was confirmed for all the categorical variables except for the zones (*p*-value > 0.05). The Mann-Whitney U test in Table 4 Part II was conducted to test if there was a difference between the groups in this studied population. Hence, with the *p*-values < 0.05, H_3_ was then confirmed.

Additionally, the mosaic plots in Figure 2 were determined; red indicates a significant negative relationship, blue indicates a significant positive relationship, and white indicates no difference. The mosaic plots indicated that the patient non-conveyance decisions occurred mainly with females in the urban area and for the age category less than 14 years old and over 59 years old. In contrast, for the males, the age category 25–44 years old had the most refusals in rural areas and was primarily associated with male Qatari and South Asian nationalities.

The binary logistic regression analysis was conducted, as shown in Table 5 and Annexed 7, to identify the determinants of patient non-conveyance decisions. Both the full variables and the backwards method were conducted. The variables listed in Table 5 and Annexed 7 are the predictors of the backward model of non-conveyance decisions. In Table 5, the significant *p*-values, Odd Ratios (OR), and positive coefficients indicated a significant positive likelihood of non-conveyance decisions when the predictors increased. These predictors were gender (Male), months (May, June, July, August, September, October, November, December), nationalities (Qatari, South Asian, Other GCC and Sub-Saharan Africa), chief complaint (Back Pain), units’ categories (Bravo and Events’ units), provisional diagnoses (as listed in Table 5), and T1 response time duration. The Odds Ratio (OR) indicates the likelihood of the patient non-conveyance decisions for every unit increase in the predictors. The coefficients indicate whether the predictor positively or negatively affects the patient non-conveyance decisions. In Table 5, for example, for the gender, the more we had male patients, the more the likelihood of patient non-conveyance increased (factor of 1.11 times higher). For patients in an urban area, their likelihood of non-conveyance decreased (negative coefficient in Annexed 7). Due to the considerable number of variables, only the variables with significant *p*-values and positive coefficients were listed in Table 5. The remaining are listed in Annexed 7.

Additionally, the AIC and Cp of Mallow’s values in Table 5 helped choose the best regression model between the full and backward variables models. Their values indicate that the backward model is the best. Hence, the Chi-square *p*-value (*p* < 0.05) indicates no significant difference between both models.

## 4. Discussion

Undeniably, the COVID-19 pandemic has affected the number of emergency medical demands, consequently affecting the number of patient non-conveyance due to potential concerns regarding the risk of infection in emergency departments [36,37]. Further, Protocol 36 of the “Pandemic” chief complaint was the most utilized protocol during emergency medical call-taking (Table 2), accounting for 16.58% (*n* = 39,447) of non-conveyance. In addition, 4.22% (*n* = 9946) of the patient non-conveyance 999 calls were diagnosed as COVID-19-related (Table 3). Recent research in Turkey highlighted that the “Stay at home” call during the COVID-19 pandemic caused a significant drop in medical and trauma ED visits, consequently increasing home mortality and morbidity risks [38]. This would increase EMS pre-hospital response activation. Another USA-based study demonstrated that although COVID-19 resulted in a significant decrease in numbers and acuity in EMS patients and emergency department admissions, the percentage of patient non-conveyance increased [37]. Furthermore, the first COVID-19 case in Qatar was declared on 28 February 2020, nine weeks after the first identified cluster in Wuhan, China [39]. Hence, the lockdown measures had been established in Qatar before the first case identification, causing overcrowding in accommodation, mainly within the workforce population often living in small shared flats and representing around 75% of Qatar’s population [39]. This resulted in a significant increase in COVID-19 cases unwilling to go to the hospital, increasing EMS demand and patient non-conveyance numbers. This would explain, firstly, the high number of patient non-conveyance cases for 999 calls with the pandemic-related complaint in Table 2 and, secondly, the high numbers of patient non-conveyance recorded from January to May in Table 1, compared to the remaining months. Further, as identified in recent reports, they might be driven by the high number of COVID-19 patients recorded in Qatar during these months, especially in 2020 and 2021 [40]. Most sporting and non-sporting events have been held within these periods (i.e.,: the Al Adaid desert challenge, the Sealine desert camping season, Lusail Moto GP circuit race) [41]. The HMCAS manages the health coverage of most of these events. Therefore, with the crowdedness and the Shamal and Easterly winds increasing during these months [42], the risk of minor and mild respiratory and Ear-Nose-Throats (ENT) diseases also sharply rises, increasing the 999 emergency demands [43].

In this study, overall, fewer than 0.16% (*n* = 549) of patient non-conveyance emergency calls were calls where the ambulance was dispatched but then cancelled. This was due to the patient’s condition no longer requiring emergency medical assistance. For 0.15% (*n* = 501) of the calls, patient non-conveyance cases were attributed to the EMD, who, according to the final medical dispatch code, recommended the patient to go to the nearest health center as emergency medical assistance was not required.

In this study, the most commonly identified protocols by the EMDs for pediatric emergency calls (age ≤ 14 years) were chief complaints with protocols 26 (sick person), 17 (falls), 30 (traumatic injuries), 16 (road traffic accidents), 11 (choking), and 6 (breathing problem) (Table 2). In the same context, the most frequent provisional diagnoses determined by the paramedics were “NSP”, “minor trauma”, “febrile illness”, “minor illness”, “respiratory”, and “gastrointestinal gastro-urinary” (GIGU) diseases (*n* = 1199) (Table 3), which corresponds to the literature [5,14,44]. Therefore, pediatric non-conveyance case follow-up is recommended, as their respiratory prognosis can worsen, and systemic problems such as sepsis can occur if not appropriately treated in a healthcare facility. Recent research in Qatar highlighted that there had been a rising trend in the last few years of Group B Streptococcal and Norovirus infections requiring hospital admissions [45,46]. Consequently, pediatric febrile non-conveyance cases should be handled cautiously by encouraging them to access primary healthcare centers [47].

Studies have also identified that the elderly’s non-conveyance calls were mainly “NSP” and were often under triaged, resulting in patient callbacks for life-threatening conditions, such as stroke [6]. Likewise, in Table 2, for the elderly non-conveyance emergency calls (age ≥ 74 years), the most utilized chief complaint protocols were 36 (pandemic), 6 (breathing problems), 26 (sick person), 10 (chest pain), 31 (unconscious), and 13 (diabetic problem). Likewise, in Table 3, the most frequently retained provisional diagnoses for elderly patients were “NSP”, “pain”, “chronic medical condition”, “respiratory diseases”, “minor illness”, and “GIGU problems”. Hence, these provisional diagnoses can be associated with pre-existing morbidities and physio-pathological conditions that may lead to the under triage of life-threatening neurological diseases. Though elderly patient non-conveyance and callback have never been explored in Qatar, vigilance would be advised to avoid under triaging critically ill elderly patients and leaving them unattended in out-of-hospital environments with ambiguous prognoses.

**Figure 3:**
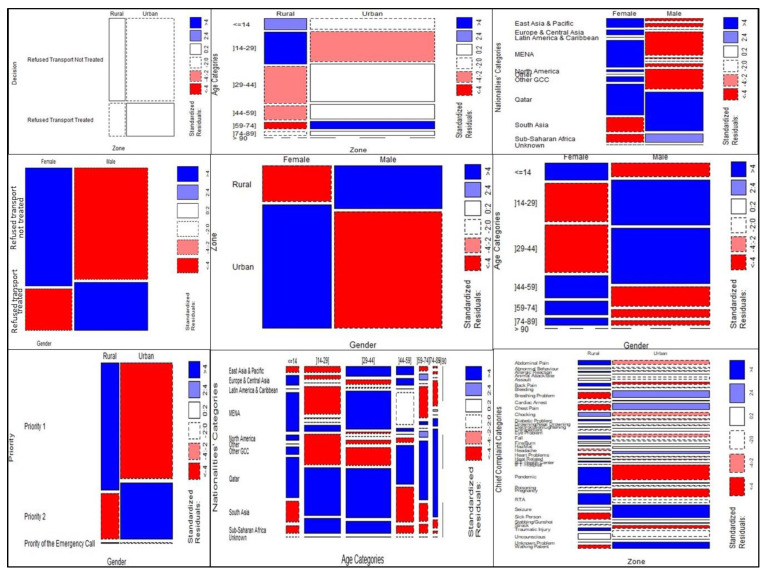
Mosaic charts of the categorical variables.

Nevertheless, as Qatar is a MENA country, ethical and cultural components were fundamental factors affecting the non-conveyance decisions. Moreover, for the non-conveyance-not-treated group’s gender and nationality in Figure 3, the non-transport decision was more highly correlated with females from MENA, including Qatar. In the case of a health emergency, the cultural component contributes to their decision not to go to hospital and gives priority to house chores and responsibilities as long as an emergency health condition is excluded. Some social research has reported on this subject [48,49]. In the same context, national reports and research revealed that most MENA females (around 70%), including Qatari, are located in the urban zones of Doha and Al-Rayaan districts [7,50]. Moreover, in Figure 3, most male patient non-conveyance cases in the rural area were Qatari and South Asians. Qatar has been developing its agricultural and manufacturing industries in the last decade, which is mainly located in rural areas, the number of South Asian laborers has increased in these rural areas, representing 75% of Qatar’s population, as have their occupational medical complaints [39,51]. Saudi Arabia has also demonstrated that medical emergency callers in rural areas often avoided travelling to the hospital by ambulance and preferred using their private cars to be able to return home more easily [5,52,53]. These results are expected considering the socio-demographic composition of the population in Qatar, with a high male predominance (72.23%) [50]. In addition, the national reports have also revealed that South Asian and Arabs nationalities have registered the most cross-border movement in Qatar during the last few years [50], [54]. Research has also explored the issues between MENA patients and healthcare systems and identified the “patient’s family demanding behavior” [55]. Middle eastern communities over-value the time with family, so when they are sick, they push the healthcare professionals to treat and release them quickly, and avoid prolonged contact with the healthcare sector [55,56].

Nonetheless, research has advocated the patient non-conveyance decisions to hospitals as a beneficial practice for health systems [14,57]. Further, managing cases with non-urgently diagnosed health problems and releasing them on scene can help reduce crowding in ED, allowing for appropriate resource utilization. In addition, providing emergency treatment for minor health issues, releasing patients on scene and encouraging them to access other primary healthcare centers helps them avoid unnecessary trips to the ED and avoid going through the long waiting process after triage [58]. The long waiting time and the poor understanding of the triage process can lead to patients’ stress and conflict with the healthcare personnel affecting healthcare delivery [58,59]. The five primary provisional diagnoses of non-conveyance are GIGU, Febrile Illness, COVID-19-related diseases, diabetic problems, and Chronic Medical Conditions. Studies show that if the paramedics on scene provide appropriate and concise health education, it can reduce the number of ED visits and 999 callbacks [7]. Reinforcing the paramedics’ health education knowledge and ensuring telephonic follow-up of non-conveyance cases would help to mitigate the potential adverse health outcomes as considered in some EMS systems [60,61,62].

Additionally, the bivariate analysis in Table 4 was conducted. Significant *p* values indicated a difference in the distribution of the patient non-transport group as per the variables in the same tables, confirming the results identified by the mosaic plots and corresponding to the socio-demographic composition in Qatar.

Finally, the backward binary logistic regression in Table 5 and Annexed 7 determined the predictors of patient non-conveyance decisions. They demonstrated that male patients had significantly low odds of non-conveyance. Besides that, patients with Qatari, South Asian, Other GCC, and Sub-Sharan Africa Nationalities had higher odds of non-conveyance compared to those with MENA, Europe & Central Asia and North American nationalities. This matches the finding of the descriptive analysis and mosaic plots. For the EMD call-taking process, every time a 999 emergency call was managed with a chief complaint of back pain (Protocol 5 in the ProQA), the patient non-conveyance likelihood increased compared to 999 emergency calls with the other chief complaints. With the free 999 emergency services, these patients most likely refused to be conveyed as they benefited from on-site intravenous painkillers or/and intramuscular anti-inflammatories, as per HMCAS CPG. They looked to avoid the long waiting time in ED in positions that might increase their pain. In other worldwide EMS services, the EMS clinician decided not to convey patients with similar complaints to hospital after on-site pain management [63]. Further, Increasing odds of patient non-conveyance were significantly associated with the provisional diagnoses of anaphylaxis, diabetic problems, febrile illnesses, and GIGU. The patients diagnosed with anaphylaxis were mainly known to have allergies. Hence, they were familiar with its treatment and felt better after receiving intramuscular Epinephrine^®^ and improving, then refusing transport to hospital. This was also demonstrated in a similar study [64]. However, leaving patients with similar provisional diagnoses in out-of-the-hospital environments without a follow-up jeopardizes their healthcare outcome. For the response time durations, the increase in T1 (The time from receiving 999 to identifying the patient’s address and the closest ambulance) increased the odds of patient non-conveyance. Conversely, the increase in T3 (The time from the CFS creation until the responding unit was assigned available) decreased the odds of patient non-conveyance.

The variables mentioned in Table 5 for models 1 and 2 are the determinants of the non-conveyance decisions, with the AIC for the backward and full variables model calculated, respectively, AIC = 250,169 and AIC = 250,200. The lowest the AIC, the better [65] There was no significant difference between the model with full variables and the backward model (Chi-square test *p*-value = 0.63).

Consequently, using the full variables model would be recommended to estimate the non-conveyance decisions based on all the explanatory determinants with the same predictor quality as the backward model (considering they have similar AIC). It will help provide a more comprehensive analysis of the non-conveyance adverse health outcomes epidemiology. 

## 5. Limitations

The data were secondary data collected from two different systems: ePCR and NJM. Therefore, the data frame was created after collecting both sources and combining the timing information from both systems. Nevertheless, identifying which calls were cancelled by the EMD was difficult as the timing of both systems often did not match. Moreover, the timing of when the response crew were with the patient was not, unfortunately, updated accurately and continuously by the EMD when reported by the field crew. This would suggest a further emphasis on changing the policies to encourage EMDs to update this timing accurately.

As these data had never been requested before, the missing timing data prevented us from further analysis by determining the amount of time specifically spent with the patient from when paramedics were at the patient’s side.

The ePCR archiving system’s limitation in identifying the healthcare personnel within the non-conveyance patients and the patient’s refusal causes prevented us from performing further analysis by measuring the level of awareness of this issue within the healthcare environment and identifying the potential rationales.

The patient non-conveyance by the EMD decision to refer the emergency caller with non-urgent chief complaints to primary healthcare centers is a newly implemented measure in HMCAS that started during the COVID-19 pandemic. Its dispatch information is captured by the NJM, which the MOI controls. This led to obtaining information with many missing values preventing us from performing a proper descriptive analysis for the non-conveyance by EMDs’ decision.

## 6. Conclusions

Patient non-conveyance to hospital decisions is a multivariable health issue. Though sometimes not concerning, considering they involve a low range of pediatric and elderly patients, they remain a serious healthcare system problem that requires further investigation. Furthermore, patients’ non-conveyance decisions strain the pre-hospital medical response resources due to responding, generally, non-urgent complaints. These decisions from patients might affect the ability of the pre-hospital healthcare system to ensure access to adequate and safe care for patients who require urgent medical attention, consequently with the potential to affect patient care outcomes. Therefore, further studies exploring the group of conveyed patients to hospital might support implementing the non-conveyance based on clinicians’ decisions and reviewing the related policies and SOPs in the HMCAS.

Hence, considering the socio-demographic diversity, the patient non-conveyance decision in Qatar and the Middle East should be explored in depth with more advanced analysis techniques. It will help understand what prompts such decisions from patients, what controls it, and how to predict it to save resources and preserve lives. It will also determine if patient education campaigns could contribute to changing this paradigm.

## Figures and Tables

**Table 1:** Basics 999 emergency calls non-conveyance statistics (*n* = 334,392).

Description	Sub-Groups	Data after Pre-Processing	
		Frequencies	Percentages (%)
Decision	Refused Transport and Not Treated	169,552	71.28
	Refused Transport and Treated	67,062	28.19
DOA		1248	0.52
Gender	Male	145,610	61.22
	Female	92,252	38.78
Zone	Urban	177,424	74.59
	Rural	60,438	25.41
Age groups (Years)	≤14	24,769	10.41
	(15–29)	68,586	28.83
	(30–44)	84,841	35.67
	(45–59)	33,252	13.98
	(60–74)	16,635	6.99
	(75–89)	8766	3.69
	≥90	1013	0.43
Years	2018 (June to December)	21,671	9.11
	2019	43,771	18.40
	2020	61,936	26.04
	2021	62,964	26.47
	2022 (January to July)	47,520	19.98
Months	April	34,325	14.43
	March	3558	14.11
	January	33,382	14.03
	May	32,396	13.62
	June	30,208	12.70
	February	27,768	11.67
	July	10,276	4.32
	December	9181	3.86
	August	6901	2.90
	November	6875	2.89
	September	6585	2.77
	October	6407	2.69
Response priorities	Priority 1	163,923	68.92
	Priority 2	73,388	30.85
	Others (Walking patients, referral, or self-dispatch)	551	0.23
Region based on Nationality	South Asia	69,849	29.37
	MENA	57,936	24.36
	Qatar	56,558	23.78
	Sub-Saharan Africa	19,302	8.12
	East Asia and the Pacific	12,286	5.17
	Europe and Central Asia	9143	3.84
	Other GCC	8863	3.73
	North America	3212	1.35
	Latin America and the Caribbean	453	0.19
	Unknown	203	0.09
	Other (i.e., officials such as United Nations)	57	0.02
Emergency Response Units	Alpha	206,338	86.75
	Bravo	19,663	8.27
	Charlie	331	0.14
	COVID	111	0.05
	Delta	505	0.21
	Event	2198	0.92
	Foxtrot	368	0.16
	Green Bus	1028	0.43
	SEM	7193	3.02
	Tango	127	0.05

**Table 2:** Emergency Call-taking chief complaints per age category (Top 15).

	Age Group Categories (Years)															
	≤14		(15–29)		(30–44)		(45–59)		(60–74)		(75–89)		≥90		Total	
	%	*n*	%	*n*	%	*N*	%	*n*	%	*n*	%	*N*	%	*n*	%	*N*
Pandemic (P 36)	1.5	3622	3.7	8779	6.1	14,584	2.4	5726	1.6	3793	1.1	2568	0.15	375	16.58	39,447
Sick Person (P 26)	1.5	3600	3.1	7431	3.4	8184	1.7	4119	1.1	2489	0.5	1161	0.04	116	11.38	27,100
RTA (P 29)	0.8	1788	4.2	9902	3.6	8645	0.9	2141	0.1	332	0	52	0	5	9.602	22,865
Breathing Problem (P 6)	0.7	1627	1.9	4569	2.3	5381	1.1	2588	0.7	1733	0.6	1393	0.06	163	7.33	17,454
Chest Pain (P 10)	0.1	292	1.1	2682	2.5	5932	1.4	3221	0.7	1697	0.4	888	0.02	64	6.19	14,776
Unconscious (P 31)	0.3	751	2	4860	2.3	5404	0.9	2057	0.4	909	0.2	433	0.02	54	6.083	14,468
Walking Patient	0.6	1530	1.8	4174	1.9	4591	0.8	1927	0.4	881	0.1	199	0	7	5.593	13,309
Abdominal Pain (P 1)	0.2	463	1.5	3512	1.8	4296	0.6	1432	0.3	629	0.1	320	0.01	27	4.5	10,679
Choking (P 11)	0.7	1649	1.4	3224	1.4	3377	0.4	837	0.1	237	0	33	0.01	12	3.935	9369
Fall (P 17)	1.1	2505	0.7	1713	0.8	1947	0.4	1047	0.2	384	0.1	161	0.01	12	3.265	7769
Seizure (P 12)	0.3	590	1.1	2597	1.1	2661	0.3	672	0.1	208	0	75	0.01	11	2.865	6814
Traumatic Injury (P 30)	0.9	2057	0.9	2036	0.8	1812	0.2	508	0.1	168	0	60	0	0	2.803	6641
Non-traumatic Back Pain (P5)	0	53	0.6	1463	1.2	2912	0.6	1333	0.2	396	0.1	132	0	5	2.652	6294
Assault (P 4)	0.1	180	1.1	2492	1.2	2803	0.2	545	0	42	0	11	0	2	2.571	6075
Heart Problems (P 19)	0.1	285	0.5	1293	0.9	2241	0.4	943	0.2	512	0.1	183	0.01	29	2.31	5486

**Table 3:** Patient non-transport provisional diagnoses per age (Top 15).

	Age Group Categories (Years)															
	≤14		(15–29)		(30–44)		(45–59)		(60–74)		(75–89)		≥90		Total	
	%	*n*	%	*n*	%	*n*	%	*n*	%	*n*	%	*n*	%	*n*	%	*n*
NSP	3.06	7268	6.14	14,600	7.49	17,822	2.83	6727	1.55	3690	0.77	1841	0.1	203	21.9	52,151
Pain	0.46	1102	4.15	9859	6.88	14,480	2.7	6425	1.18	2816	0.5	1194	0.04	99	15.9	35,975
Minor Trauma	2.05	4869	5.67	13,488	4.5	11,876	1.21	2876	0.35	838	0.11	258	0.01	30	13.9	34,235
Neurological	0.25	587	2.58	6138	2.99	7102	1.19	2826	0.44	1036	0.18	419	0.01	30	7.64	18,138
GIGU	0.5	1199	2.25	5340	2.95	7013	1.04	2480	0.46	1095	0.25	598	0.02	47	7.47	17,772
Minor Illness	0.71	1687	1.36	3240	1.86	4427	0.83	1976	0.53	1253	0.35	827	0.04	103	5.68	13,513
Respiratory	0.63	1497	1.39	3305	1.83	4356	0.79	1886	0.38	907	0.37	868	0.05	108	5.44	12,927
Trauma	0.55	1311	1.6	3810	1.58	3762	0.43	1026	0.1	247	0.03	59	0	1	4.29	10,216
Febrile Illness	0.86	2056	1.04	2470	1.4	3305	0.47	1108	0.28	665	0.18	417	0.03	78	4.26	10,099
COVID-19 Related	0.48	1134	0.81	1920	1.8	4183	0.7	1667	0.3	720	0.12	289	0.01	33	4.22	9946
Diabetic Problem	0.04	89	0.22	524	0.42	987	0.4	939	0.41	968	0.23	550	0.01	23	1.73	4080
CMC	0.04	86	0.13	312	0.3	703	0.33	783	0.47	1112	0.38	894	0.1	152	1.75	4042
Cardiovascular	0.01	30	0.16	378	0.47	1112	0.49	1166	0.33	787	0.15	361	0.01	32	1.62	3866
Burns	0.18	433	0.36	860	0.04	917	0.14	325	0.04	93	0.01	16	0	0	0.77	2644
Allergic Reaction	0.16	830	0.2	483	0.22	537	0.09	205	0.03	73	0.01	16	0	3	0.71	2147

**Table 4:** Bivariate analyses.

Part I: Chi-Square Test of Patient Non-Conveyance Decisions Groups and Other Groups of Variables:				
Variables	Subgroups	Patient Non-conveyance decisions		Chi-square
		Refused Transport and Not Treated	Refused Transport and Treated	
		observed (expected)	observed (expected)	
Zone	Rural	43,058 (43,047.61)	17,016 (17,039.68)	X^2^ = 0.01, df = 1, *p*-value = 0.91
	Urban	126,494 (126,504.39)	50,046 (50,035.61)	
Gender	Male	101,361 (103,793.24)	43,214 (41,052.79)	X^2^ = 438.34, df = 1, *p*-value < 2.2 × 10^−16^
	Female	68,191 (65,758.76)	23,848 (26,009.21)	
Year	2018 (June to December)	15,578 (15,447.45)	5955 (6109.84)	X^2^ = 74.93, df = 4, *p*-value = 2.06 × 10^−15^
	2019	31,054 (31,200.70)	12,492 (12,340.64)	
	2020	44,747 (44,149.01)	16,907 (17,462.02)	
	2021	44,158 (44,881.79)	18,455 (17,751.85)	
	2022 (January to July)	34,015 (33,873.05)	13,253 (13,397.62)	
Response priorities	Priority 1	122,323 (116,847.04)	40,441 (46,215.89)	X^2^ = 3157.8, df = 2, *p*-value = 2.2 × 10^−16^
	Priority 2	46,830 (52,312.19)	26,469 (20,690.76)	
	Other response priorities	399 (392.76)	152 (155.34)	
**Part II: The Mann-Whitney U Test (Wilcoxon Rank Sum Test) for the Non-Conveyance:**				
			W-values	*p*-values
Patient non-conveyance decisions	Months		5,562,400,046	<2.2 × 10^−16^
	Nationalities Categories		5,419,676,164	2.2 × 10^−8^
	Provisional diagnoses categories		5,182,154,299	<2.2 × 10^−16^
	Response units categories		6,102,256,789	<2.2 × 10^−16^
	Age categories		5,580,476,590	3.59 × 10^−13^
	Duration from CFS creation to assigned available		5,647,879,250	0.01
	Duration from CFS creation to pending dispatch		6,196,555,806	<2.2 × 10^−16^
	Duration from CFS pending to active dispatch		5,950,198,517	<2.2 × 10^−16^

**Table 5:** Backward Regression analysis model outcome.

Part (I) Variables with Positive Coefficients in the Backward Model							
Variables		Sub-categories		Coefficients	OR	*p*-value	95% CI
Gender		Male		0.11	1.11	<0.001	0.09, 0.13
Years		2019		0.15	1.17	<0.001	0.11, 0.20
		2020		0.40	1.50	<0.001	0.35, 0.46
		2021		0.56	1.75	<0.001	0.51, 0.61
		2022		0.48	1.62	<0.001	0.43, 0.54
Months		August		0.21	1.23	<0.001	0.14, 0.28
		December		0.26	1.30	<0.001	0.20, 0.32
		July		0.12	1.13	<0.001	0.06, 0.17
		June		0.08	1.08	<0.001	0.04, 0.12
		May		0.08	1.08	<0.001	0.04, 0.12
		November		0.31	1.36	<0.001	0.24, 0.38
		October		0.21	1.23	<0.001	0.14, 0.28
		September		0.31	1.36	<0.001	0.24, 0.38
Nationalities categories		Other GCC		0.15	1.16	<0.001	0.08, 0.22
		Qatar		0.18	1.19	<0.001	0.13, 0.23
		South Asia		0.25	1.28	<0.001	0.20, 0.30
		Sub-Saharan Africa		0.24	1.27	<0.001	0.19, 0.30
Chief complaints (Call-taking protocols)		Back Pain (Protocol 5)		0.21	1.23	<0.001	0.14, 0.27
Units’ categories		Bravo		1.3	3.72	<0.001	1.3, 1.4
		Event		0.24	1.27	<0.001	0.14, 0.34
Provisional diagnoses categories		Anaphylaxis		1.3	3.61	<0.001	0.86, 1.7
		Burns		1.0	2.82	<0.001	0.86, 1.2
		Cardiovascular		0.27	1.31	0.002	0.10, 0.44
		CMC		0.48	1.62	<0.001	0.32, 0.65
		Diabetic Problem		0.93	2.54	<0.001	0.76, 1.1
		Epistaxis		0.68	1.98	<0.001	0.48, 0.89
		Febrile Illness		0.94	2.57	<0.001	0.79, 1.1
		GIGU		0.91	2.49	<0.001	0.76, 1.1
		HazMat		0.41	1.51	<0.001	0.19, 0.63
		Heat-Related		1.4	3.93	<0.001	1.2, 1.6
		Minor Trauma		1.3	3.79	<0.001	1.2, 1.5
		Neurological		0.78	2.19	<0.001	0.63, 0.94
		Not Recorded		1.9	6.67	<0.001	0.98, 2.8
		Pain		0.98	2.66	<0.001	0.83, 1.1
		Respiratory		0.80	2.23	<0.001	0.65, 0.96
		Shock		2.0	7.56	<0.001	1.6, 2.5
		Sick Person		0.75	2.11	0.004	0.24, 1.2
		Trauma		1.1	3.14	<0.001	0.98, 1.3
Response time durations		T1 (“Pending_Creation”)		3.06 × 10^−3^	1.00	0.003	0.00, 0.01
**Part (II) Regression Analysis Models’ Comparison**							
	Resid. Df	Resid. Dev	Df	DV	Cp	AIC	Pr (>Chi)
Model 1	236,498	249,941	0	−1.71	250,173	250,200	0.63
Model 2	236,501	249,943	0	0	250,173	250,169	
Deviance Residuals:			Min	1Q	Median	3Q	Max
	Model 1		−1.944	−0.861	−0.5248	1.0219	2.8513
	Model 2		−1.945	−0.861	−0.5246	1.0217	2.8508

Model 1 (Full variables model): Decision~Zone + Priority + Gender + Year + Month + AgeCategories + NAT_Categories + ChiefComplaint_CAT + Unit_Categories + P.Diagnosis_CAT + CFS Creation_Pending dispatch_MIN (T1) + Pending _Active dispatch_MIN (T2) + CFS creation_Assigned available_MIN (T3). Model 2 (Backward model): Decision~Zone + Gender + Year + Month + AgeCategories + NAT_Categories +ChiefComplaint_CAT + Unit_Categories + P.Diagnosis_CAT + CFS creation_Assigned available_MIN (T3) + CFS Creation_Pending dispatch_MIN (T1).

## Data Availability

The anonymous data supporting the findings of this study are held by the corresponding author and are available for review upon request.

## Supplementary Materials

The following supporting information can be downloaded at: https://www.mdpi.com/article/10.3390/ijerph20146404/s1. Annexed 1: Zones categories. Annexed 2: Emergency medical dispatch chief complaints codes. Annexed 3: Responding units’ categorization. Annexed 4: Provisional diagnoses classification. Annexed 5: Non-conveyance to hospital decision classification as per HMCAS patient non-conveyance classification system. Annexed 6: Ages classification. Annexed 7: Regression analysis of backward model variables with negative coefficients.
