# Supplementary material for: Epidemiological Determinants of Patient Non-Conveyance to the Hospital in an Emergency Medical Service Environment
Source: Int J Environ Res Public Health. 2023 Jul 20;20(14):6404. doi: 10.3390/ijerph20146404 (PMC10379159; doi:10.3390/ijerph20146404)
Supplement: Supplementary file 1 [file ijerph-20-06404-s001.zip › ijerph-2201684-supplementary.pdf]

Supplementary files

Annexe 1: Zones categories

Zone Number	*ZoneCategory*	*Zone Number*	*Zone Category*
0	Urban	49	Urban
1	Urban	50	Urban
2	Urban	51	Urban
3	Urban	52	Urban
4	Urban	53	Urban
5	Urban	54	Urban
6	Urban	55	Urban
7	Urban	56	Urban
10	Urban	57	Urban
11	Urban	58	Urban
12	Urban	60	Urban
13	Urban	61	Urban
14	Urban	62	Urban
15	Urban	63	Urban
16	Urban	64	Urban
17	Urban	65	Urban
18	Urban	66	Urban
19	Urban	67	Urban
20	Urban	68	Urban
21	Urban	69	Urban
22	Urban	70	Rural
23	Urban	71	Rural
24	Urban	72	Rural
25	Urban	73	Rural
26	Urban	74	Rural
27	Urban	75	Rural
28	Urban	76	Rural
29	Urban	77	Rural
30	Urban	78	Rural
31	Urban	79	Rural
32	Urban	80	Rural
33	Urban	81	Rural
34	Urban	82	Rural
35	Urban	83	Rural
36	Urban	84	Rural
37	Urban	85	Rural
38	Urban	86	Rural
39	Urban	90	Rural
40	Urban	91	Rural
41	Urban	92	Rural
42	Urban	93	Rural
43	Urban	94	Rural
44	Urban	95	Rural
45	Urban	96	Rural
46	Urban	97	Rural
47	Urban	98	Rural
48	Urban	0	Urban

Annexe 2: Emergency medical dispatch chief complaint codes

DISPATCH CODE	CHIEF COMPLAINT	DISPATCH CODE	CHIEF COMPLAINT	DISPATCH CODE	CHIEF COMPLAINT	DISPATCH CODE	CHIEF COMPLAINT	DISPATCH CODE	CHIEF COMPLAINT	DISPATCH CODE	CHIEF COMPLAINT
**01A01**	Abdominal Pain	07D05	Fire/Burn	122800	Seizure	150801	Electrocution/Lightening	28C01G	Strock	29D03U	RTA
**01A02**	Abdominal Pain	07D05E	Fire/Burn	122900	Seizure	150802	Electrocution/Lightening	28C01H	Strock	29D03V	RTA
**01C00**	Abdominal Pain	07D05F	Fire/Burn	123200	Seizure	150803	Electrocution/Lightening	28C01J	Strock	29D04	RTA
**01C01**	Abdominal Pain	08B01	HazMat	123201	Seizure	15C01E	Electrocution/Lightening	28C01K	Strock	29D04V	RTA
**01C02**	Abdominal Pain	08B01C	HazMat	123202	Seizure	15C01L	Electrocution/Lightening	28C01L	Strock	29D04Y	RTA
**01C03**	Abdominal Pain	08B01G	HazMat	123300	Seizure	15D02E	Electrocution/Lightening	28C01M	Strock	29D05	RTA
**01C04**	Abdominal Pain	08B01M	HazMat	123400	Seizure	15D04E	Electrocution/Lightening	28C01U	Strock	29D05U	RTA
**01C05**	Abdominal Pain	08B01U	HazMat	123501	Seizure	15D07E	Electrocution/Lightening	28C01X	Strock	29D05V	RTA
**01C06**	Abdominal Pain	08C01	HazMat	123502	Seizure	15D08E	Electrocution/Lightening	28C01Y	Strock	29D05X	RTA
**01D00**	Abdominal Pain	08C01C	HazMat	123601	Seizure	15D08L	Electrocution/Lightening	28C01Z	Strock	29D05Y	RTA
**01D01**	Abdominal Pain	08C01G	HazMat	123602	Seizure	15D09E	Electrocution/Lightening	28C02C	Strock	29D06	RTA
**02A01**	Allergic reaction	08C01M	HazMat	123603	Seizure	15E01E	Electrocution/Lightening	28C02F	Strock	29D06U	RTA
**02A01I**	Allergic reaction	08C01U	HazMat	123604	Seizure	160800	Eye problem	28C02G	Strock	29D06V	RTA
**02A01M**	Allergic reaction	08D01G	HazMat	123605	Seizure	160900	Eye problem	28C02H	Strock	29D06Y	RTA
**02A02**	Allergic reaction	08D02	HazMat	123700	Seizure	161100	Eye problem	28C02J	Strock	29D07	RTA
**02B00**	Allergic reaction	08D02C	HazMat	123800	Seizure	16A01	Eye problem	28C02K	Strock	29D07U	RTA
**02B00M**	Allergic reaction	08D02G	HazMat	123801	Seizure	16A02	Eye problem	28C02L	Strock	29D07V	RTA
**02B01**	Allergic reaction	08D02M	HazMat	123802	Seizure	16A03	Eye problem	28C02U	Strock	29D08	RTA
**02B01I**	Allergic reaction	08D03C	HazMat	124000	Seizure	16B00	Eye problem	28C02X	Strock	29D08V	RTA
**02B01M**	Allergic reaction	08D03G	HazMat	124200	Seizure	16B01	Eye problem	28C02Y	Strock	29D09	RTA
**02C01**	Allergic reaction	08D03M	HazMat	124201	Seizure	16D01	Eye problem	28C03C	Strock	29D09U	RTA
**02C01I**	Allergic reaction	08D03U	HazMat	124300	Seizure	170102	Fall	28C03D	Strock	29D09V	RTA
**02C01M**	Allergic reaction	08D04	HazMat	124301	Seizure	170203	Fall	28C03F	Strock	29D09Y	RTA
**02C02**	Allergic reaction	08D04B	HazMat	124302	Seizure	170501	Fall	28C03G	Strock	29O01	RTA
**02C02I**	Allergic reaction	08D04C	HazMat	124600	Seizure	170502	Fall	28C03H	Strock	29O01U	RTA
**02C02M**	Allergic reaction	08D04G	HazMat	124700	Seizure	170503	Fall	28C03J	Strock	29O01V	RTA
**02D01**	Allergic reaction	08D04M	HazMat	124800	Seizure	170506	Fall	28C03K	Strock	29O01Y	RTA
**02D01I**	Allergic reaction	08D04T	HazMat	124900	Seizure	170702	Fall	28C03L	Strock	30A01	Traumatic injury
**02D01M**	Allergic reaction	08D04U	HazMat	125400	Seizure	170704	Fall	28C03U	Strock	30A02	Traumatic injury
**02D02**	Allergic reaction	08D05	HazMat	125402	Seizure	170706	Fall	28C03X	Strock	30A03	Traumatic injury
**02D02I**	Allergic reaction	08D05B	HazMat	125500	Seizure	170709	Fall	28C03Y	Strock	30B00	Traumatic injury
**02D02M**	Allergic reaction	08D05C	HazMat	125600	Seizure	170902	Fall	28C04C	Strock	30B01	Traumatic injury
**02D03**	Allergic reaction	08D05G	HazMat	125800	Seizure	170908	Fall	28C04D	Strock	30B02	Traumatic injury
**02D04**	Allergic reaction	08D05M	HazMat	126200	Seizure	170909	Fall	28C04F	Strock	30B03	Traumatic injury
**02E01**	Allergic reaction	08D05U	HazMat	126300	Seizure	171101	Fall	28C04G	Strock	30D01	Traumatic injury
**03A01**	Animal attack/bite	08D06C	HazMat	126301	Seizure	171201	Fall	28C04H	Strock	30D02	Traumatic injury
**03A02**	Animal attack/bite	08D06G	HazMat	126303	Seizure	171202	Fall	28C04J	Strock	30D03	Traumatic injury
**03A03**	Animal attack/bite	08D06T	HazMat	126401	Seizure	171203	Fall	28C04K	Strock	30D04	Traumatic injury
**03A04**	Animal attack/bite	09B01a	Cardiac arrest	126500	Seizure	171205	Fall	28C04L	Strock	30D05	Traumatic injury
**03B00**	Animal attack/bite	09B01b	Cardiac arrest	126501	Seizure	171301	Fall	28C04U	Strock	31A01	Uncounscious
**03B01**	Animal attack/bite	09B01c	Cardiac arrest	126502	Seizure	171303	Fall	28C04X	Strock	31A02	Uncounscious

**03B02**	Animal attack/bite	09B01e	Cardiac arrest	126601	Seizure	171304	Fall	28C04Y	Strock	31A03	Uncounscious
**03B03**	Animal attack/bite	09B01f	Cardiac arrest	126602	Seizure	171305	Fall	28C05C	Strock	31C00	Uncounscious
**03D02**	Animal attack/bite	09D01	Cardiac arrest	126803	Seizure	171406	Fall	28C05D	Strock	31C01	Uncounscious
**03D03**	Animal attack/bite	09D02	Cardiac arrest	126900	Seizure	171601	Fall	28C05F	Strock	31C02	Uncounscious
**03D04**	Animal attack/bite	09D02a	Cardiac arrest	127000	Seizure	171602	Fall	28C05G	Strock	31C03	Uncounscious
**03D05**	Animal attack/bite	09D02e	Cardiac arrest	127200	Seizure	171603	Fall	28C05J	Strock	31D01	Uncounscious
**03D06**	Animal attack/bite	09D02x	Cardiac arrest	127300	Seizure	171604	Fall	28C05K	Strock	31D02	Uncounscious
**03D07**	Animal attack/bite	09E01	Cardiac arrest	127400	Seizure	171605	Fall	28C05L	Strock	31D03	Uncounscious
**03D08**	Animal attack/bite	09E02	Cardiac arrest	127600	Seizure	171606	Fall	28C05U	Strock	31D04	Uncounscious
**03D09**	Animal attack/bite	09E03	Cardiac arrest	127700	Seizure	171608	Fall	28C05X	Strock	31D05	Uncounscious
**04A01A**	Assault	09E04	Cardiac arrest	127701	Seizure	171609	Fall	28C05Y	Strock	31E00	Uncounscious
**04A02A**	Assault	10A01	Chest pain	127800	Seizure	171701	Fall	28C06G	Strock	31E01	Uncounscious
**04A02S**	Assault	10C00	Chest pain	127901	Seizure	171703	Fall	28C06J	Strock	32B01	Unknown problem
**04A02T**	Assault	10C01	Chest pain	129100	Seizure	171705	Fall	28C06L	Strock	32B02	Unknown problem
**04A03A**	Assault	10C03	Chest pain	129500	Seizure	171706	Fall	28C06X	Strock	32B03	Unknown problem
**04A03S**	Assault	10D01	Chest pain	129600	Seizure	171709	Fall	28C06Y	Strock	32B04	Unknown problem
**04B00A**	Assault	10D02	Chest pain	129800	Seizure	171800	Fall	28C07C	Strock	32D00	Unknown problem
**04B00S**	Assault	10D03	Chest pain	129801	Seizure	171900	Fall	28C07D	Strock	32D01	Unknown problem
**04B01A**	Assault	10D04	Chest pain	129802	Seizure	172000	Fall	28C07F	Strock	33A01P	IFT-Health Center
**04B01S**	Assault	10D05	Chest pain	12A01E	Seizure	173001	Fall	28C07G	Strock	33A01T	IFT-Health Center
**04B02A**	Assault	110101	Chocking	12A02	Seizure	173002	Fall	28C07H	Strock	33A02T	IFT-Health Center
**04B03A**	Assault	110102	Chocking	12A03	Seizure	17A01	Fall	28C07J	Strock	33A03T	IFT-Health Center
**04B03S**	Assault	110103	Chocking	12A03E	Seizure	17A01A	Fall	28C07L	Strock	33C00T	IFT-Health Center
**04B03T**	Assault	110200	Chocking	12A04	Seizure	17A01G	Fall	28C07X	Strock	33C01T	IFT-Health Center
**04D00T**	Assault	1102601	Chocking	12A04E	Seizure	17A01J	Fall	28C07Y	Strock	33C02T	IFT-Health Center
**04D01A**	Assault	110500	Chocking	12A05	Seizure	17A01P	Fall	28C08C	Strock	33C03T	IFT-Health Center
**04D02A**	Assault	110501	Chocking	12A05E	Seizure	17A02	Fall	28C08G	Strock	33C04T	IFT-Health Center
**04D03A**	Assault	110502	Chocking	12B00E	Seizure	17A02A	Fall	28C08J	Strock	33C05T	IFT-Health Center
**04D03S**	Assault	110601	Chocking	12B01	Seizure	17A02E	Fall	28C08L	Strock	33C06T	IFT-Health Center
**04D04A**	Assault	110602	Chocking	12B01E	Seizure	17A02G	Fall	28C08X	Strock	33C07T	IFT-Health Center
**04D05A**	Assault	110603	Chocking	12C00	Seizure	17A02P	Fall	28C08Y	Strock	33D00T	IFT-Health Center
**05A01**	Back pain	110700	Chocking	12C01	Seizure	17A03	Fall	28C09C	Strock	360002	Pandemic
**05A02**	Back pain	110800	Chocking	12C01E	Seizure	17A03G	Fall	28C09D	Strock	36A01A	Pandemic
**05C00**	Back pain	110802	Chocking	12C02	Seizure	17A04	Fall	28C09G	Strock	36A01C	Pandemic
**05C01**	Back pain	110901	Chocking	12C02E	Seizure	17A04G	Fall	28C09J	Strock	36A01S	Pandemic
**05C02**	Back pain	110902	Chocking	12C03	Seizure	17B00	Fall	28C09K	Strock	36A02A	Pandemic
**05C03**	Back pain	110903	Chocking	12C03E	Seizure	17B00G	Fall	28C09L	Strock	36A02B	Pandemic
**05C04**	Back pain	111100	Chocking	12C04	Seizure	17B00P	Fall	28C09X	Strock	36A02C	Pandemic
**05D01**	Back pain	111200	Chocking	12C05	Seizure	17B01	Fall	28C10Z	Strock	36A02S	Pandemic
**06C01**	Breathing problem	111512	Chocking	12C05E	Seizure	17B01G	Fall	28C11C	Strock	36A03A	Pandemic
**06C01A**	Breathing problem	111521	Chocking	12C07	Seizure	17B01J	Fall	28C11G	Strock	36A03B	Pandemic
**06C01E**	Breathing problem	111526	Chocking	12C07E	Seizure	17B01P	Fall	28C11L	Strock	36A03C	Pandemic
**06C01O**	Breathing problem	111527	Chocking	12D01	Seizure	17B02	Fall	28C11U	Strock	36A03S	Pandemic
**06C02**	Breathing problem	11160	Chocking	12D01E	Seizure	17B02G	Fall	28C11X	Strock	36A04	Pandemic
**06C02A**	Breathing problem	111602	Chocking	12D02	Seizure	17B02P	Fall	28C11Y	Strock	36C00A	Pandemic
**06C02O**	Breathing problem	111702	Chocking	12D02E	Seizure	17B03	Fall	28C12G	Strock	36C00B	Pandemic

**06D01**	Breathing problem	111901	Chocking	12D03	Seizure	17B03E	Fall	28C12U	Strock	36C00C	Pandemic
**06D01A**	Breathing problem	111902	Chocking	12D03E	Seizure	17B03G	Fall	29A00V	RTA	36C00S	Pandemic
**06D01E**	Breathing problem	111903	Chocking	12D04	Seizure	17B03J	Fall	29A00X	RTA	36C01A	Pandemic
**06D01O**	Breathing problem	112001	Chocking	12D04E	Seizure	17B03P	Fall	29A01	RTA	36C01B	Pandemic
**06D02**	Breathing problem	112003	Chocking	13A01	Diabetic problem	17B04	Fall	29A01V	RTA	36C01C	Pandemic
**06D02A**	Breathing problem	1120031	Chocking	13C00	Diabetic problem	17B04G	Fall	29A01Y	RTA	36C01S	Pandemic
**06D02E**	Breathing problem	1120032	Chocking	13C01	Diabetic problem	17B04P	Fall	29A02	RTA	36C02A	Pandemic
**06D02O**	Breathing problem	112101	Chocking	13C01C	Diabetic problem	17D01	Fall	29A02U	RTA	36C02B	Pandemic
**06D03**	Breathing problem	112102	Chocking	13C02	Diabetic problem	17D01E	Fall	29A02V	RTA	36C02C	Pandemic
**06D03A**	Breathing problem	112202	Chocking	13C02C	Diabetic problem	17D02	Fall	29A02X	RTA	36C02S	Pandemic
**06D03E**	Breathing problem	112204	Chocking	13C03	Diabetic problem	17D03	Fall	29A02Y	RTA	36C03A	Pandemic
**06D03O**	Breathing problem	112206	Chocking	13C03C	Diabetic problem	17D04	Fall	29B00	RTA	36C03B	Pandemic
**06D04**	Breathing problem	11A01C	Chocking	13D01	Diabetic problem	17D04G	Fall	29B00U	RTA	36C03C	Pandemic
**06D04A**	Breathing problem	11A01F	Chocking	140800	Drowning/Near drowning	17D04P	Fall	29B01	RTA	36C03S	Pandemic
**06D04E**	Breathing problem	11A01M	Chocking	14A01	Drowning/Near drowning	17D05	Fall	29B01U	RTA	36C04A	Pandemic
**06D04O**	Breathing problem	11A01O	Chocking	14A01S	Drowning/Near drowning	17D05G	Fall	29B01V	RTA	36C04B	Pandemic
**06D05**	Breathing problem	11A01U	Chocking	14B01	Drowning/Near drowning	17D06	Fall	29B01X	RTA	36C04C	Pandemic
**06E00**	Breathing problem	11D01C	Chocking	14B01D	Drowning/Near drowning	180102	Headache	29B01Y	RTA	36C04S	Pandemic
**06E00O**	Breathing problem	11D01F	Chocking	14B01S	Drowning/Near drowning	180400	Headache	29B02	RTA	36C05A	Pandemic
**06E01**	Breathing problem	11D01M	Chocking	14C01	Drowning/Near drowning	180502	Headache	29B02U	RTA	36C05B	Pandemic
**06E01A**	Breathing problem	11D01O	Chocking	14D01	Drowning/Near drowning	180503	Headache	29B02V	RTA	36C05C	Pandemic
**06E01O**	Breathing problem	11D01U	Chocking	14D01D	Drowning/Near drowning	18A01	Headache	29B02Y	RTA	36C05S	Pandemic
**07A01**	Fire/Burn	11D02F	Chocking	14D02	Drowning/Near drowning	18B00	Headache	29B03	RTA	36D00A	Pandemic
**07A01E**	Fire/Burn	11D02M	Chocking	14D04	Drowning/Near drowning	18B01	Headache	29B03U	RTA	36D00B	Pandemic
**07A01F**	Fire/Burn	11D02O	Chocking	14D05	Drowning/Near drowning	18C00	Headache	29B03V	RTA	36D00C	Pandemic
**07A02**	Fire/Burn	11D02U	Chocking	14D05D	Drowning/Near drowning	18C00Y	Headache	29B03X	RTA	36D01A	Pandemic
**07A03**	Fire/Burn	11E01	Chocking	14D05S	Drowning/Near drowning	18C01	Headache	29B03Y	RTA	36D01B	Pandemic
**07A03E**	Fire/Burn	11E01C	Chocking	14E01	Drowning/Near drowning	18C01C	Headache	29B04	RTA	36D01C	Pandemic
**07A04**	Fire/Burn	11E01F	Chocking	14E02	Drowning/Near drowning	18C01D	Headache	29B04V	RTA	36D01S	Pandemic
**07A05**	Fire/Burn	11E01M	Chocking	150101	Electrocution/Lightening	18C01F	Headache	29B04X	RTA	36D02A	Pandemic
**07B00**	Fire/Burn	11E01O	Chocking	150102	Electrocution/Lightening	18C01G	Headache	29B04Y	RTA	36D02B	Pandemic
**07B01**	Fire/Burn	11E01U	Chocking	150104	Electrocution/Lightening	18C01H	Headache	29B05	RTA	36D02C	Pandemic
**07B01E**	Fire/Burn	120010	Seizure	150106	Electrocution/Lightening	18C01J	Headache	29B05U	RTA	36D02S	Pandemic
**07B02**	Fire/Burn	1200101	Seizure	150108	Electrocution/Lightening	18C01K	Headache	29B05V	RTA	36D03A	Pandemic
**07B02E**	Fire/Burn	1200201	Seizure	150110	Electrocution/Lightening	18C01L	Headache	29B05X	RTA	36D03B	Pandemic
**07B02F**	Fire/Burn	1200202	Seizure	150201	Electrocution/Lightening	18C01U	Headache	29B05Y	RTA	36D03C	Pandemic
**07B02W**	Fire/Burn	120700	Seizure	150301	Electrocution/Lightening	18C01X	Headache	29D00Y	RTA	36D03S	Pandemic
**07C01F**	Fire/Burn	120800	Seizure	150302	Electrocution/Lightening	18C01Y	Headache	29D01a	RTA	36D04A	Pandemic
**07C02**	Fire/Burn	121000	Seizure	150304	Electrocution/Lightening	18C01Z	Headache	29D01b	RTA	36D04B	Pandemic
**07C02E**	Fire/Burn	121100	Seizure	150305	Electrocution/Lightening	18C02	Headache	29D01d	RTA	36D04C	Pandemic
**07C02F**	Fire/Burn	121200	Seizure	150311	Electrocution/Lightening	18C02C	Headache	29D01e	RTA	36D04S	Pandemic
**07C03**	Fire/Burn	121300	Seizure	150313	Electrocution/Lightening	18C02D	Headache	29D01f	RTA	37B01	IFT-Hospital
**07C03E**	Fire/Burn	121400	Seizure	150314	Electrocution/Lightening	18C02G	Headache	29D01h	RTA	37B01S	IFT-Hospital
**07C04**	Fire/Burn	121500	Seizure	150503	Electrocution/Lightening	18C02H	Headache	29D02k	RTA	37B02	IFT-Hospital
**07C04E**	Fire/Burn	121600	Seizure	150504	Electrocution/Lightening	18C02J	Headache	29D02l	RTA	37B02S	IFT-Hospital

**07D01**	Fire/Burn	121602	Seizure	150505	Electrocution/Lightening	18C02K	Headache	29D02m	RTA	37C02	IFT-Hospital
**07D01E**	Fire/Burn	121900	Seizure	150601	Electrocution/Lightening	18C02L	Headache	29D02n	RTA	37C04	IFT-Hospital
**07D01F**	Fire/Burn	122100	Seizure	150602	Electrocution/Lightening	18C02M	Headache	29D02o	RTA	37C04B	IFT-Hospital
**07D02**	Fire/Burn	122101	Seizure	150606	Electrocution/Lightening	18C02U	Headache	29D02p	RTA	37C05	IFT-Hospital
**07D02F**	Fire/Burn	122102	Seizure	150608	Electrocution/Lightening	18C02X	Headache	29D02q	RTA	37C05A	IFT-Hospital
**07D04**	Fire/Burn	122300	Seizure	150611	Electrocution/Lightening	18C02Y	Headache	29D02r	RTA	AS10X	Walking patient
**07D04E**	Fire/Burn	122400	Seizure	150612	Electrocution/Lightening	18C03C	Headache	29D02s	RTA	AS10Y	Walking patient
**07D04F**	Fire/Burn	122500	Seizure	150613	Electrocution/Lightening	18C03D	Headache	29D02t	RTA	AS12X	Walking patient
		122600	Seizure	150700	Electrocution/Lightening	18C03F	Headache	29D03	RTA	AS12Y	Walking patient

DISPATCH CODE	Chief Complaint	Dispatch Code	Chief Complaint	Dispatch Code	Chief Complaint	Dispatch Code	Chief Complaint	Dispatch Code	Chief Complaint	Dispatch Code	Chief Complaint
**18C03G**	Headache	190800	Heart problems	23C02I	Poisoning	26A00	Sick person			AS14X	Walking patient
**18C03H**	Headache	191700	Heart problems	23C02V	Poisoning	26A01	Sick person			AS15X	Walking patient
**18C03J**	Headache	191800	Heart problems	23C03A	Poisoning	26A02	Sick person			AS16X	Walking patient
**18C03K**	Headache	192100	Heart problems	23C03I	Poisoning	26A03	Sick person			AS17X	Walking patient
**18C03L**	Headache	193400	Heart problems	23C04A	Poisoning	26A04	Sick person			AS17Y	Walking patient
**18C03U**	Headache	19A01	Heart problems	23C04I	Poisoning	26A05	Sick person			AS18X	Walking patient
**18C03X**	Headache	19A02	Heart problems	23C06A	Poisoning	26A06	Sick person			AS18Y	Walking patient
**18C03Y**	Headache	19C00	Heart problems	23C06I	Poisoning	26A07	Sick person			AS19X	Walking patient
**18C03Z**	Headache	19C01	Heart problems	23C07A	Poisoning	26A08	Sick person			AS19Y	Walking patient
**18C04C**	Headache	19C02	Heart problems	23C07I	Poisoning	26A09	Sick person			AS1X	Walking patient
**18C04D**	Headache	19C03	Heart problems	23C07V	Poisoning	26A10	Sick person			AS1Y	Walking patient
**18C04F**	Headache	19C04	Heart problems	23C07W	Poisoning	26A11	Sick person			AS20X	Walking patient
**18C04G**	Headache	19C06	Heart problems	23C08	Poisoning	26A12	Sick person			AS21X	Walking patient
**18C04H**	Headache	19C07	Heart problems	23D01A	Poisoning	26B00	Sick person			AS22X	Walking patient
**18C04J**	Headache	19D01	Heart problems	23D01I	Poisoning	26B01	Sick person			AS23X	Walking patient
**18C04K**	Headache	19D02	Heart problems	23D02A	Poisoning	26C00	Sick person			AS24X	Walking patient
**18C04L**	Headache	19D03	Heart problems	23D02I	Poisoning	26C01	Sick person			AS25X	Walking patient
**18C04U**	Headache	19D04	Heart problems	23D03A	Poisoning	26C02	Sick person			AS25Y	Walking patient
**18C04X**	Headache	200010	Heat related	23D03I	Poisoning	26C03	Sick person			AS26X	Walking patient
**18C04Y**	Headache	200011	Heat related	23O01A	Poisoning	26C04	Sick person			AS26Y	Walking patient
**18C04Z**	Headache	200999	Heat related	24A01	Pregnancy	26D00	Sick person			AS29X	Walking patient
**18C05D**	Headache	203800	Heat related	24B01	Pregnancy	26D01	Sick person			AS29Y	Walking patient
**18C05G**	Headache	203901	Heat related	24B01M	Pregnancy	26O02	Sick person			AS2X	Walking patient
**18C05H**	Headache	204000	Heat related	24B02	Pregnancy	26O03	Sick person			AS2Y	Walking patient
**18C05J**	Headache	20A01C	Heat related	24C01	Pregnancy	26O04	Sick person			AS30X	Walking patient
**18C05K**	Headache	20A01H	Heat related	24C02	Pregnancy	26O05	Sick person			AS30Y	Walking patient
**18C05L**	Headache	20B00H	Heat related	24C03	Pregnancy	26O06	Sick person			AS31X	Walking patient
**18C05U**	Headache	20B01H	Heat related	24C03M	Pregnancy	26O07	Sick person			AS31Y	Walking patient
**18C05X**	Headache	20B02C	Heat related	24D03	Pregnancy	26O08	Sick person			AS32X	Walking patient
**18C05Y**	Headache	20B02H	Heat related	24D03M	Pregnancy	26O09	Sick person			AS32Y	Walking patient
**18C06G**	Headache	20C01H	Heat related	24D04	Pregnancy	26O10	Sick person			AS3X	Walking patient
**18C06J**	Headache	20D01C	Heat related	24D05	Pregnancy	26O11	Sick person			AS4X	Walking patient
**18C06L**	Headache	20D01H	Heat related	24D05M	Pregnancy	26O12	Sick person			AS4Y	Walking patient
**18C06X**	Headache	20D02H	Heat related	24O01	Pregnancy	26O13	Sick person			AS5X	Walking patient

**18C06Y**	Headache	210601	Bleeding	250103	Abnormal behaviour	26O14	Sick person	AS6X	Walking patient
**18C07D**	Headache	2121243	Bleeding	250111	Abnormal behaviour	26O15	Sick person	AS6Y	Walking patient
**18C07F**	Headache	21A01M	Bleeding	250203	Abnormal behaviour	26O16	Sick person	AS7X	Walking patient
**18C07G**	Headache	21A01T	Bleeding	250401	Abnormal behaviour	26O17	Sick person	AS7Y	Walking patient
**18C07K**	Headache	21A02M	Bleeding	25A01	Abnormal behaviour	26O18	Sick person	AS9X	Walking patient
**18C07L**	Headache	21A02T	Bleeding	25A01B	Abnormal behaviour	26O19	Sick person	AS9Y	Walking patient
**18C07X**	Headache	21B00M	Bleeding	25A01V	Abnormal behaviour	26O20	Sick person	ASX	Walking patient
**18C07Y**	Headache	21B00T	Bleeding	25A01W	Abnormal behaviour	26O21	Sick person	ASY	Walking patient
**19002**	Heart problems	21B01M	Bleeding	25A02	Abnormal behaviour	26O22	Sick person		
**190100**	Heart problems	21B01T	Bleeding	25A02V	Abnormal behaviour	26O23	Sick person		
**190200**	Heart problems	21B02M	Bleeding	25B02	Abnormal behaviour	26O24	Sick person		
		21B02T	Bleeding	25B02B	Abnormal behaviour	26O26	Sick person		
		21B03M	Bleeding	25B02V	Abnormal behaviour	26O27	Sick person		
		21B03T	Bleeding	25B02W	Abnormal behaviour	26O28	Sick person		
		21B04M	Bleeding	25B03	Abnormal behaviour	27B02G	Stabbing/Gunshot		
		21B04T	Bleeding	25B03B	Abnormal behaviour	27B02I	Stabbing/Gunshot		
		21C01M	Bleeding	25B03T	Abnormal behaviour	27B02P	Stabbing/Gunshot		
		21C01T	Bleeding	25B03V	Abnormal behaviour	27B02S	Stabbing/Gunshot		
		21C02M	Bleeding	25B03W	Abnormal behaviour	27B02Y	Stabbing/Gunshot		
		21C03M	Bleeding	25B04	Abnormal behaviour	27B04S	Stabbing/Gunshot		
		21C03T	Bleeding	25B04V	Abnormal behaviour	27D01S	Stabbing/Gunshot		
		21D02M	Bleeding	25B05	Abnormal behaviour	27D01Y	Stabbing/Gunshot		
		21D02T	Bleeding	25B06	Abnormal behaviour	27D02S	Stabbing/Gunshot		
		21D03M	Bleeding	25B06B	Abnormal behaviour	27D03S	Stabbing/Gunshot		
		21D03T	Bleeding	25B06V	Abnormal behaviour	27D03Y	Stabbing/Gunshot		
		21D04M	Bleeding	25B06W	Abnormal behaviour	27D04S	Stabbing/Gunshot		
		21D04T	Bleeding	25D01	Abnormal behaviour	27D04Y	Stabbing/Gunshot		
		21D05M	Bleeding	25D01B	Abnormal behaviour	27D05S	Stabbing/Gunshot		
		21D05T	Bleeding	25D01V	Abnormal behaviour	27D05Y	Stabbing/Gunshot		
		22A01	Entrapement	25D02	Abnormal behaviour	27D06S	Stabbing/Gunshot		
		22A01M	Entrapement	25D03	Abnormal behaviour	28A01G	Strock		
		22B00	Entrapement	25D03B	Abnormal behaviour	28A01J	Strock		
		22B01	Entrapement	25D03V	Abnormal behaviour	28A01L	Strock		
		22B01B	Entrapement	25D03W	Abnormal behaviour	28A01U	Strock		
		22B02	Entrapement	25D04	Abnormal behaviour	28A01X	Strock		
		22B02A	Entrapement			28C01C	Strock		
		22B03	Entrapement			28C01D	Strock		
		22B03A	Entrapement			28C01F	Strock		
		22D01	Entrapement						
		22D01A	Entrapement						
		22D01M	Entrapement						
		22D01X	Entrapement						
		22D01Y	Entrapement						
		22D03A	Entrapement						
		22D04	Entrapement						

	22D04A	Entrapement
	22D04M	Entrapement
	22D04X	Entrapement
	22D05	Entrapement
	22D05A	Entrapement
	22D05M	Entrapement
	230102	Poisoning
	230701	Poisoning
	23B01I	Poisoning
	23B01V	Poisoning
	23C01A	Poisoning
	23C01I	Poisoning
	23C01V	Poisoning
	23C02	Poisoning
	23C02A	Poisoning

Annexe 3: Responding unit categorisation

RESPONDING UNIT	TYPE	RESPONDING UNIT	TYPE	RESPONDING UNIT	TYPE	RESPONDING UNIT	TYPE	RESPONDING UNIT	TYPE
**1.015**	Alpha	A5.025	Alpha	b4.1	Bravo	E-AL WAJBA	Event	HAZMAT 1	SEM
**2.011**	Alpha	A5.026	Alpha	BRV2.1	Bravo	E-Asayel	Event	HAZMAT 2	SEM
**2.02**	Alpha	A5.03	Alpha	BRV4.1	Bravo	E-ASPIRE	Event	HAZMAT 4	SEM
**4.015**	Alpha	A5.04	Alpha	BRV8.1	Bravo	ECAMEL	Event	HAZMAT 4.01	SEM
**4.10**	Alpha	A5.05	Alpha	BRV8.2	Bravo	E-CAMEL	Event	HAZMAT 4.1	SEM
**5.001**	Alpha	A5.06	Alpha	BRV8.3	Bravo	e-camel3	Event	HAZMAT 5	SEM
**5.11**	Alpha	A5.07	Alpha	BRV8.4	Bravo	E-CAMPING 1	Event	HAZMAT4.1	SEM
**A 7.011**	Alpha	A5.08	Alpha	BRV8.5	Bravo	e-cherch	Event	MIR 2.01	SEM
**A?**	Alpha	A5.09	Alpha	BRV8.6	Bravo	e-church	Event	MIR 2.1	SEM
**A1.01**	Alpha	A5.1	Alpha	BRV8.7	Bravo	e-cup4	Event	MIR 6.1	SEM
**A1.010**	Alpha	A5.10	Alpha	BRV8.8	Bravo	E-CYCLING 2	Event	MIR4.01	SEM
**A1.011**	Alpha	A5.11	Alpha	GOLF81	Bravo	E-D7.1	Event	MIR4.1	SEM
**A1.014**	Alpha	A5.12	Alpha	GOLF82	Bravo	E-D71	Event	MIR6.1	SEM
**A1.02**	Alpha	A5.13	Alpha	CDO	Charlie	E-DELTA 10	Event	MIR7.01	SEM
**A1.03**	Alpha	A5.14	Alpha	CH01	Charlie	E-DRAG 1	Event	MIR7.1	SEM
**A1.04**	Alpha	A5.15	Alpha	CH02	Charlie	E-DRAG 2	Event	T 6.1	Tango
**A1.05**	Alpha	A5.16	Alpha	CH03	Charlie	E-DRAG 3	Event	T1.10	Tango
**A1.06**	Alpha	A5.17	Alpha	CH04	Charlie	e-drag race2	Event	T1.11	Tango
**A1.07**	Alpha	A5.18	Alpha	CH05	Charlie	edrag1	Event	T1.12	Tango
**A1.08**	Alpha	A5.19	Alpha	CH06	Charlie	E-ENDURANCE	Event	T1.13	Tango
**A1.09**	Alpha	A5.2	Alpha	CH07	Charlie	E-EXHIBITION	Event	T1.14	Tango
**A1.1**	Alpha	A5.20	Alpha	CH08	Charlie	E-exibition	Event	T1.15	Tango
**A1.10**	Alpha	A5.21	Alpha	CH09	Charlie	E-fes 1	Event	T1.16	Tango
**A1.11**	Alpha	A5.22	Alpha	CH11	Charlie	EFESTIVAL	Event	T1.17	Tango
**A1.12**	Alpha	A5.23	Alpha	CH2.01	Charlie	e-festival	Event	T1.18	Tango
**A1.13**	Alpha	A5.24	Alpha	CH3.01	Charlie	E-FESTIVAL 1	Event	T1.19	Tango
**A1.14**	Alpha	A5.25	Alpha	CH4.01	Charlie	E-FESTIVAL 2	Event	T1.2	Tango
**A1.15**	Alpha	A5.26	Alpha	CH5.01	Charlie	E-FIFA 1	Event	T1.20	Tango
**A1.16**	Alpha	A5.27	Alpha	CH7.01	Charlie	E-FIFA 1.1	Event	T1.21	Tango
**A1.17**	Alpha	A5.3	Alpha	CHLF101	Charlie	E-FOOTBALL 1	Event	T1.22	Tango
**A1.18**	Alpha	A5.4	Alpha	CHLF102	Charlie	e-halal	Event	T1.23	Tango
**A1.19**	Alpha	A5.5	Alpha	CHLF103	Charlie	e-hayat	Event	T1.24	Tango
**A1.2**	Alpha	A5.6	Alpha	COHORT 4	Charlie	E-HORSE 1	Event	T1.27	Tango
**A1.20**	Alpha	A5.7	Alpha	COHORT 6	Charlie	ekatara	Event	T1.28	Tango
**A1.21**	Alpha	A5.8	Alpha	CVC	Charlie	e-katara	Event	T1.29	Tango
**A1.22**	Alpha	A5.9	Alpha	LF101	Charlie	e-katara1	Event	T1.3	Tango
**A1.3**	Alpha	A6.01	Alpha	LF102	Charlie	Elousil	Event	T1.5	Tango
**A1.4**	Alpha	A6.010	Alpha	LF103	Charlie	e-lusail	Event	T1.7	Tango
**A1.5**	Alpha	A6.015	Alpha	OSC. E	Charlie	E-mahamyl	Event	T1.8	Tango
**A1.6**	Alpha	A6.02	Alpha	Qatar 1	COVID	E-mahanil	Event	T1.9	Tango
**A1.7**	Alpha	A6.03	Alpha	QATAR 1.4	COVID	e-mahaseel	Event	T2.1	Tango
**A1.8**	Alpha	A6.04	Alpha	QATAR 2	COVID	e-majaheem	Event	T2.2	Tango
**A1.9**	Alpha	A6.05	Alpha	Qatar 3	COVID	E-MARATHON 1	Event	T2.3	Tango
**A2.0.2**	Alpha	A6.06	Alpha	QATAR 4.3	COVID	E-MARATHON 3	Event	T4.1	Tango
**A2.01**	Alpha	A6.07	Alpha	QATAR 7	COVID	emariot	Event	T4.2	Tango
**A2.010**	Alpha	A6.08	Alpha	qatar1	COVID	entretient	Event	T4.3	Tango
**A2.011**	Alpha	A6.09	Alpha	QATAR1.02	COVID	E-ORYX 2	Event	T4.4	Tango
**A2.012**	Alpha	A6.1	Alpha	QATAR1.1	COVID	E-ORYX 3	Event	T4.5	Tango
**A2.013**	Alpha	A6.10	Alpha	QATAR1.2	COVID	E-ORYX 4	Event	T4.6	Tango
**A2.014**	Alpha	A6.11	Alpha	QATAR1.8	COVID	E-ORYX 5	Event	T4.7	Tango
**A2.016**	Alpha	A6.12	Alpha	QATAR10	COVID	E-ORYX 6	Event	T5.1	Tango
**A2.02**	Alpha	A6.13	Alpha	qatar3	COVID	E-Qatar 1	Event	T5.2	Tango
**A2.03**	Alpha	A6.14	Alpha	qatar4	COVID	E-Qatar 3	Event	T5.3	Tango
**A2.04**	Alpha	A6.15	Alpha	QATAR5.4	COVID	E-QATAR 4	Event	T5.4	Tango
**A2.05**	Alpha	A6.18	Alpha	QATAR5.5	COVID	E-QATAR 4.3	Event	T5.5	Tango
**A2.06**	Alpha	A6.2	Alpha	QATAR5.6	COVID	eqatara	Event	T7.1	Tango
**A2.07**	Alpha	A6.3	Alpha	QATAR5.7	COVID	E-QMMF	Event	T7.2	Tango
**A2.08**	Alpha	A6.4	Alpha	QATAR6	COVID	e-rail4	Event		
**A2.09**	Alpha	A6.5	Alpha	QATAR6.1	COVID	E-SCHOOL	Event		
**A2.1**	Alpha	A6.6	Alpha	QATAR7	COVID	e-shanglrila	Event		
**A2.10**	Alpha	A6.7	Alpha	QATAR8	COVID	E-shangrila	Event		
**A2.11**	Alpha	A6.8	Alpha	QATAR9	COVID	E-SHAQAB 1	Event		
**A2.12**	Alpha	A6.9	Alpha	qx1	COVID	E-SHERATON	Event		
**A2.13**	Alpha	A7.01	Alpha	D 1	Delta	ESOUK WAKEF	Event		
**A2.14**	Alpha	A7.010	Alpha	D 10	Delta	E-SOUK WKA	Event		
**A2.15**	Alpha	A7.02	Alpha	D 2	Delta	ESOUKWAKEF	Event		
**A2.16**	Alpha	A7.03	Alpha	D 2.1	Delta	E-souq	Event		
**A2.17**	Alpha	A7.04	Alpha	D 3	Delta	E-SPARTAN 2	Event		

**A2.18**	Alpha	A7.05	Alpha	D 4	Delta	E-spring	Event
**A2.19**	Alpha	A7.06	Alpha	D 4.1	Delta	E-UNIVERSITY	Event
**A2.2**	Alpha	A7.07	Alpha	D 5	Delta	event	Event
**A2.20**	Alpha	A7.08	Alpha	D 5.1	Delta	e-village	Event
**A2.21**	Alpha	A7.09	Alpha	D 6	Delta	E-WAJBA	Event
**A2.3**	Alpha	A7.1	Alpha	D 6.1	Delta	Exhibition	Event
**A2.4**	Alpha	A7.10	Alpha	D 7	Delta	exibition unit	Event
**A2.5**	Alpha	A7.11	Alpha	D C 4	Delta	FEVER-CLINIC 1	Event
**A2.6**	Alpha	A7.12	Alpha	D10	Delta	FEVER-CLINIC 2	Event
**A2.7**	Alpha	A7.13	Alpha	D2	Delta	FEVER-CLINIC 3	Event
**A2.8**	Alpha	A7.14	Alpha	D4	Delta	HMC1	Event
**A2.9**	Alpha	A7.15	Alpha	D5.1	Delta	katara	Event
**A4.01**	Alpha	A7.16	Alpha	D7	Delta	ORYX 2	Event
**A4.010**	Alpha	A7.17	Alpha	D9	Delta	Rehersal 1	Event
**A4.011**	Alpha	A7.18	Alpha	ajyal event	Event	samla2	Event
**A4.012**	Alpha	a7.19	Alpha	almajahin	Event	VACCINE 1	Event
**A4.013**	Alpha	A7.2	Alpha	aq1	Event	VACCINE 1.01	Event
**A4.014**	Alpha	a7.20	Alpha	assayel1	Event	VACCINE 2	Event
**A4.015**	Alpha	a7.21	Alpha	at7.23	Event	VACCINE 3	Event
**A4.016**	Alpha	a7.22	Alpha	at7.24	Event	vaccine1.01	Event
**A4.02**	Alpha	A7.24	Alpha	drag1	Event	F1.01	Foxtrot
**A4.03**	Alpha	A7.25	Alpha	Duhail 3	Event	F1.012	Foxtrot
**A4.04**	Alpha	A7.26	Alpha	E - A7.12	Event	F1.013	Foxtrot
**A4.05**	Alpha	A7.27	Alpha	E - A7.13	Event	F1.02	Foxtrot
**A4.06**	Alpha	A7.3	Alpha	E - A7.14	Event	F1.03	Foxtrot
**A4.07**	Alpha	A7.4	Alpha	E - A7.15	Event	F1.05	Foxtrot
**A4.08**	Alpha	A7.5	Alpha	E - A7.16	Event	F1.06	Foxtrot
**A4.09**	Alpha	A7.6	Alpha	E - A7.17	Event	F1.1	Foxtrot
**A4.1**	Alpha	A7.7	Alpha	E - A7.18	Event	F1.10	Foxtrot
**A4.10**	Alpha	A7.8	Alpha	E - A7.19	Event	F1.11	Foxtrot
**A4.11**	Alpha	A7.9	Alpha	E - A7.20	Event	F1.12	Foxtrot
**A4.12**	Alpha	A8.1	Alpha	E - A7.21	Event	F1.13	Foxtrot
**A4.13**	Alpha	A8.2	Alpha	E - A7.22	Event	F1.14	Foxtrot
**A4.14**	Alpha	A8.3	Alpha	E- A7.15	Event	F1.15	Foxtrot
**A4.15**	Alpha	A8.4	Alpha	E CAMEL	Event	F1.16	Foxtrot
**A4.16**	Alpha	DRIVING 1	Alpha	E Camel2	Event	F1.17	Foxtrot
**A4.17**	Alpha	DRIVING 2	Alpha	e exhibition	Event	F1.2	Foxtrot
**A4.18**	Alpha	DRIVING 3	Alpha	E EZDAN 4	Event	F1.3	Foxtrot
**A4.19**	Alpha	DRIVING 4	Alpha	E HORSE2	Event	F1.4	Foxtrot
**A4.2**	Alpha	DRIVING 5	Alpha	e katara	Event	F1.5	Foxtrot
**A4.20**	Alpha	DRIVING 6	Alpha	E KATARA3	Event	F1.6	Foxtrot
**A4.21**	Alpha	DRIVING 7	Alpha	E LOUSIL	Event	F1.7	Foxtrot
**A4.22**	Alpha	driving2	Alpha	e lusail	Event	F1.8	Foxtrot
**A4.23**	Alpha	driving4	Alpha	e mahaseel	Event	F1.9	Foxtrot
**A4.24**	Alpha	LVQ01	Alpha	E RAS GAZ	Event	F4.1	Foxtrot
**A4.25**	Alpha	LVQ1	Alpha	E SCHOOL	Event	F6.01	Foxtrot
**A4.26**	Alpha	LVQ2	Alpha	e souq wakra	Event	F6.1	Foxtrot
**A4.27**	Alpha	LVQ3	Alpha	E Vaccin 1.01	Event	F7.01	Foxtrot
**A4.28**	Alpha	POOL UNIT 1	Alpha	E-A7.12	Event	F7.1	Foxtrot
**A4.3**	Alpha			E-A7.12 4x4 Unit	Event	RETRIEVAL 1	Foxtrot
**A4.4**	Alpha			e-a7.13	Event	RETRIEVAL 2	Foxtrot
**A4.5**	Alpha			E-A7.13 4X4 Unit	Event	gb 9	Green bus
**A4.6**	Alpha			E-A7.14 4X4 Unit	Event	GB0	Green bus
**A4.7**	Alpha			e-a7.15	Event	GB5.1	Green bus
**A4.8**	Alpha			e-a7.16	Event	gb6	Green bus
**A4.9**	Alpha			E-A7.17	Event	GB7.1	Green bus
**A5.00**	Alpha			E-A7.18	Event	GB8.1	Green bus
**A5.001**	Alpha			E-A7.22	Event	GREEN BUS 2	Green bus
**A5.01**	Alpha			E-A712	Event	GREEN BUS ZERO	Green bus
**A5.010**	Alpha			E-A713	Event	GREENBUS 0	Green bus
**A5.011**	Alpha			E-A714	Event	GREENBUS 6	Green bus
**A5.012**	Alpha			E-A715	Event	mike2	Other
**A5.013**	Alpha			E-A716	Event	mile1	Other

**A5.014**	Alpha	E-A717	Event	PRODUCTION MIR	Other
**A5.015**	Alpha	E-A718	Event	T-HAZMAT5	Other
**A5.016**	Alpha	E-A719	Event	T-LOGISTIC5	Other
**A5.017**	Alpha	E-A720	Event	X1A	Other
**A5.019**	Alpha	E-A721	Event	XI4	Other
**A5.02**	Alpha	E-A722	Event	HAZM 1	SEM
**A5.021**	Alpha	E-AFC 11	Event	HAZM 2	SEM
**A5.023**	Alpha	E-AFC 6	Event	HAZM 3	SEM
**A5.024**	Alpha	E-AL NASHAAB 2	Event	HAZM2	SEM

Annexe 4: Provisional diagnoses classification

PROVISIONAL DIAGNOSIS	CLASSIFICATION	PROVISIONAL DIAGNOSIS	CLASSIFICATION
**ALLERGIES**	Allergic reaction	CVA	Neurological
**OTHER : ALLERGIC REACTION**	Allergic reaction	Neurological : Acute Stroke < 8 Hours	Neurological
**OTHER : ANAPHYLAXIS**	Anaphylaxis	Neurological : Headache	Neurological
**ANIMAL BITE**	Animal Bite	Neurological : Psychiatric Event	Neurological
**AIRWAY BURNS- THERMALDSAD**	Burns	Neurological : Reduced Level of Conciousness Unkno	Neurological
**BURNS**	Burns	Neurological : Reduced Level of Conciousness Unknown Cause	Neurological
**BURNS : CHEMICAL**	Burns	Neurological : Seizure	Neurological
**BURNS : ELECTRICAL**	Burns	Neurological : Stroke > 8 hours	Neurological
**BURNS : INHALATION**	Burns	Neurological : Syncope	Neurological
**BURNS : THERMAL**	Burns	Neurological : Transient Ischemic Attack	Neurological
**CARDIAC ARREST**	Cardiac arrest-Medical	Seizures	Neurological
**CARDIAC PROBLEMS**	Cardiovascular	Non-specific problems	NonSpecificProblems
**CARDIOVASCULAR : ACS OTHER**	Cardiovascular	Nothing Abnormal Detected	NonSpecificProblems
**CARDIOVASCULAR : ACS STEMI**	Cardiovascular	Other : Nothing Abnormal Detected	NonSpecificProblems
**CARDIOVASCULAR : ACUTE PULMONARY EDEMA**	Cardiovascular	Non-trauma back pain	NonTraumaticBackPain
**CARDIOVASCULAR : ANGINA PECTORIS**	Cardiovascular	Not Recorded	Not Recorded
**CARDIOVASCULAR : ATRIAL FIBRILLATION**	Cardiovascular	OBS GYN : Breech Presentation	OBS GYN
**CARDIOVASCULAR : CONGESTIVE HEART FAILURE**	Cardiovascular	OBS GYN : Delivery	OBS GYN
**CARDIOVASCULAR : HYPERTENSIVE EMERGENCY**	Cardiovascular	OBS GYN : Eclampsia	OBS GYN
**CARDIOVASCULAR : NARROW COMPLEX BRADYARRHYTHMIA**	Cardiovascular	OBS GYN : Ectopic pregnancy	OBS GYN
**CARDIOVASCULAR : NARROW COMPLEX TACHYARRHYTHMIA**	Cardiovascular	OBS GYN : Hemorrhage	OBS GYN
**CARDIOVASCULAR : PERICARDITIS**	Cardiovascular	OBS GYN : Placenta Abruptio	OBS GYN
**CARDIOVASCULAR : WIDE COMPLEX BRADYARRHYTHMIA**	Cardiovascular	OBS GYN : Placenta Previa	OBS GYN
**CARDIOVASCULAR : WIDE COMPLEX TACHYARRHYTHMIA**	Cardiovascular	OBS GYN : Post Delivery Care	OBS GYN
**HYPERTENSIVE EMERGENCY**	Cardiovascular	OBS GYN : Pre Term Labor	OBS GYN
**OTHER : EXACERBATION OF CHRONIC MEDICAL CONDITION**	Chronic Medical Condition	OBS GYN : Pre-eclampsia	OBS GYN
**OTHER : MEDICAL DEVICE FAILURE**	Chronic Medical Condition	OBS GYN : Umbilical Cord Prolapse	OBS GYN
**OTHER : COMBATIVE PATIENT**	Combative Patient	Other : Pain Unknown Cause	Pain
**OTHER : CONFIRMED COVID 19**	COVID19 related	Other : Pain, Unknown Cause	Pain
**OTHER : SUSPECTED COVID 19**	COVID19 related	Pain - non-trauma	Pain
**ENDOCRINE : DIABETIC KETOACIDOSIS**	Diabetic problem	Other: Parental concern	Parental concern
**ENDOCRINE : HYPERGLYCEMIA**	Diabetic problem	Asthma	Respiratory
**ENDOCRINE : HYPOGLYCEMIA**	Diabetic problem	Recreational drugs / Alcohol OD	Respiratory
**HYPOGLYCEMIA**	Diabetic problem	Respiratory : Asthma	Respiratory
**DOA**	DOA	Respiratory : Complete FBAO	Respiratory
**OTHER : MEDICAL OBVIOUS DEATH/DOA**	DOA	Respiratory : COPD	Respiratory
**TRAUMA : OBVIOUS DEATH/DOA**	DOA	Respiratory : Croup	Respiratory
**OTHER : ELECTRICAL INJURY**	Electrocution	Respiratory : Epiglottitis	Respiratory
**OTHER : HYPERKALEMIA**	Endocrinology	Respiratory : Hyperventilation Syndrome	Respiratory
**OTHER : NOSE BLEED**	Epistaxix	Respiratory : Lower Respiratory Infection	Respiratory
**HYPERTHERMIA**	Febrile Illness	Respiratory : Lower Respiratory Infrection	Respiratory
**OTHER : FEBRILE ILLLNESS**	Febrile Illness	Respiratory : Partial FBAO	Respiratory
**ABDOMINAL PAIN**	GIGU	Respiratory : Pleurisy	Respiratory
**ABDOMINAL PROBLEM**	GIGU	Respiratory : Pneumothorax Medical	Respiratory
**GENITO -URINARY PROBLEMS**	GIGU	Respiratory : Pulmonary Edema	Respiratory

**GI GU : ACUTE ABDOMEN**	GIGU	Respiratory : Upper AIrway Swelling	Respiratory
**GI GU : CONSTIPATION**	GIGU	Respiratory : Upper Respiratory Infection	Respiratory
**GI GU : GASTROENTIRITIS**	GIGU	Shock : Distributive/Septic/Anaphylaxis	Shock
**GI GU : HEMATURIA**	GIGU	Shock : Hypovolemic	Shock
**GI GU : HERNIA**	GIGU	Shock : Obstructive	Shock
**GI GU : LIVER FAILURE**	GIGU	Dizziness	Sick person
**GI GU : RENAL COLIC**	GIGU	Headache	Sick person
**GI GU : UPPER GI BLEED**	GIGU	Malaise	Sick person
**GI GU : URINARY TRACT INFECTION**	GIGU	Medical Emergencies	Sick person
**HAZMAT : ABSORPTION**	HazMat	Envenomation	Toxicology
**HAZMAT : INGESTION**	HazMat	Environmental : Envenomation	Toxicology
**HAZMAT : INHALATION**	HazMat	Toxicological : Opioid	Toxicology
**HAZMAT : RADIATION**	HazMat	Toxicological : Organophosphate	Toxicology
**ENVIRONMENTAL : HEAT ILLNESS**	Heat related	Toxicological : Other	Toxicology
**ENVIRONMENTAL : HYPOTHERMIA**	Heat related	Toxicological : Sedative	Toxicology
**HEAT STROKE**	Heat related	Toxicological : Unknown Substance	Toxicology
**NON-EMERGENCY TRANSPORT**	IFT	Major Musculoskeletal Injury	Trauma
**OTHER : CARE BY MEDICAL ESCORT TEAM**	IFT	Trauma - Thoracic	Trauma
**TRANSPORT : IFT/PTS**	IFT	Trauma : Abdominal	Trauma
**TRANSPORT : RETRIEVAL**	IFT	Trauma : Amputation	Trauma
**OTHER: BASIC CHECK-UP**	Minor illness	Trauma : Avulsion/Degloving	Trauma
**OTHER: MINOR ILLNESS (UNSPECIFIED)**	Minor illness	Trauma : Blast Injury	Trauma
**MINOR JOINT INJURY**	Minor trauma	Trauma : Blunt Trauma	Trauma
**MINOR MUSCULOSKELETAL INJURY**	Minor trauma	Trauma : Brain Injury	Trauma
**MINOR TRAUMA**	Minor trauma	Trauma : Closed Fracture	Trauma
**NON-TRAUMA MUSCULOSKELETAL PAIN**	Minor trauma	Trauma : Dislocation	Trauma
**OTHER: MINOR INJURY (UNSPECIFIED)**	Minor trauma	Trauma : Femur Fracture(s)	Trauma
**TRAUMA - SOFT TISSUE INJURY/BRUISING/ABRASION**	Minor trauma	Trauma : Head Injury	Trauma
**TRAUMA - SPRAIN / STRAIN**	Minor trauma	Trauma : Major Hemorrhage	Trauma
**TRAUMA : LACERATION**	Minor trauma	Trauma : Pelvic fracture	Trauma
**TRAUMA : SOFT TISSUE INJURY/BRUISING/ABRASION**	Minor trauma	Trauma : Penetrating Trauma	Trauma
**TRAUMA : SPRAIN / STRAIN**	Minor trauma	Trauma : Spinal Injury	Trauma
**TRAUMA PAIN**	Minor trauma	Trauma : Thoracic	Trauma
**ENVIRONMENTAL : SUBMERSION**	Near Drowning	Trauma - Obvious Death/DOA	TraumaUndeniableDeath

Annexe 5: Non-conveyance to hospital decision classification as per HMCAS non-conveyance classification system

DECISION	CODES
**REFUSED TRANSPORT-TREATED AT SCENE**	Refused transport Treated
**TREATED AT SCENE - NOT TRANSPORTED**	Refused transport Treated

Annexe 6: Ages classification

AGE CATEGORIES
**00-14**
**15-29**
**30-44**
**45-59**
**60-74**
**75-89**
**≥90**

Annexe 7: Regression analysis backward model variables with a negative coefficients

Variables	Sub-categories	Coefficients	OR	p-value	95% CI
**Zone**	Urban	-0.07	0.93	<0.001	-0.10, -0.05
**Age categories**	≤14	-0.72	0.49	<0.001	-0.76, -0.68
	(45-59)	-0.06	0.94	<0.001	-0.09, -0.03
	(60-74)	-0.21	0.81	<0.001	-0.25, -0.17
	(75-89)	-0.23	0.8	<0.001	-0.29, -0.17
	>90	-0.22	0.8	0.008	-0.38, -0.06
**Nationalities categories**	Europe & Central Asia	-0.13	0.88	<0.001	-0.19, -0.06
	MENA	0.16	1.17	<0.001	0.11, 0.21
	North America	-0.10	0.91	0.048	-0.19, 0.00
**Chief complaints (EMD Call-taking protocols)**	Abnormal Behaviour (P 25)	-1.6	0.20	<0.001	-1.7, -1.5
	Allergic Reaction (P 02)	-0.32	0.72	<0.001	-0.47, -0.17
	Animal Attack/Bite (P 03)	-0.72	0.48	<0.001	-1.0, -0.41
	Assault (P 04)	-1.2	0.30	<0.001	-1.3, -1.1
	Bleeding (P 26)	-0.59	0.55	<0.001	-0.70, -0.49
	Breathing Problem (P 06)	-0.54	0.58	<0.001	-0.60, -0.48
	Cardiac Arrest (P 09)	-0.91	0.40	<0.001	-1.1, -0.69
	Chest Pain (P 10)	-0.74	0.48	<0.001	-0.79, -0.68
	Chocking (P 11)	-1.2	0.29	<0.001	-1.3, -1.2
	Diabetic Problem (P 13)	-0.67	0.51	<0.001	-0.77, -0.57
	Drowning/Near Drowning (14)	-1.7	0.18	<0.001	-2.4, -1.0
	Electrocution/Lightening (P 15)	-0.80	0.45	<0.001	-1.0, -0.57
	Entrapment (P 22)	-1.2	0.31	<0.001	-1.4, -0.88
	Eye Problem (P 16)	-0.88	0.41	<0.001	-1.1, -0.69
	Fall (P 17)	-0.58	0.56	<0.001	-0.66, -0.51
	Fire/Burn (P 07)	-0.31	0.73	<0.001	-0.43, -0.20
	HazMat (P 08)	-0.34	0.71	<0.001	-0.51, -0.16
	Headache (P 18)	-0.12	0.88	0.001	-0.20, -0.05
	Heart Problems (P 19)	-0.90	0.4	<0.001	-0.99, -0.82
	Heat-Related (P 20)	-0.39	0.68	<0.001	-0.52, -0.26
	IFT-Health Center (P 33)	-1.5	0.22	<0.001	-1.7, -1.3
	Pandemic (P 36)	-0.80	0.45	<0.001	-0.85, -0.75
	Poisoning (P 23)	-1.4	0.25	<0.001	-1.7, -1.1
	Pregnancy (P24)	-0.74	0.48	<0.001	-0.94, -0.54
	RTA (P 29)	-1.3	0.28	<0.001	-1.3, -1.2
	Seizure (P12)	-1.3	0.28	<0.001	-1.4, -1.2
	Sick Person (P 26)	-0.63	0.53	<0.001	-0.68, -0.58
	Stabbing/Gunshot (P 27)	-1.7	0.18	<0.001	-2.2, -1.2
	Strock (P 28)	-1.0	0.36	<0.001	-1.2, -0.83
	Traumatic Injury (P 30)	-0.45	0.64	<0.001	-0.52, -0.38
	Uncounscious (P 31)	-0.84	0.43	<0.001	-0.90, -0.77
	Unknown Problem (P 32)	-0.89	0.41	<0.001	-1.1, -0.72
	Walking Patient	-0.83	0.44	<0.001	-0.89, -0.77
**Unit_Categories**	Delta	-0.93	0.39	<0.001	-1.2, -0.67
	Foxtrot	-0.54	0.59	<0.001	-0.81, -0.26
	Green bus	-1.8	0.16	<0.001	-2.1, -1.6
	SEM	-0.77	0.46	<0.001	-0.84, -0.70
	Tango	-1.1	0.33	<0.001	-1.7, -0.56
**Provisional diagnoses categories**	Minor Illness	-0.43	0.65	<0.001	-0.59, -0.27
	Non Specific Problems	-0.47	0.62	<0.001	-0.63, -0.32
	Toxicology	-0.46	0.63	<0.001	-0.71, -0.21
**Response time durations**	T3: ("Assigned_Creation")	(-)1.73*10-4	1.00	<0.001	0.00, 0.00
